# Author Correction: Coherent electric field control of orbital state of a neutral nitrogen-vacancy center

**DOI:** 10.1038/s41467-024-50127-0

**Published:** 2024-07-19

**Authors:** Hodaka Kurokawa, Keidai Wakamatsu, Shintaro Nakazato, Toshiharu Makino, Hiromitsu Kato, Yuhei Sekiguchi, Hideo Kosaka

**Affiliations:** 1 Quantum Information Research Center, Institute of Advanced Sciences, Yokohama National University, 79-5 Tokiwadai, Hodogaya, Yokohama 240-8501 Japan; 2 Department of Physics, Graduate School of Engineering Science, Yokohama National University, 79-5 Tokiwadai, Hodogaya, Yokohama 240-8501 Japan; 3 Advanced Power Electronics Research Center, National Institute of Advanced Industrial Science and Technology, 1-1-1 Umezono, Tsukuba, Ibaraki 305-8568 Japan

**Keywords:** Qubits, Atom optics, Electronic structure of atoms and molecules

Correction to: *Nature Communications* 10.1038/s41467-024-47973-3, published online 13 May 2024

In this article the affiliation details for Hideo Kosaka were incorrectly given as Quantum Information Research Center, Institute of Advanced Sciences, Yokohama National University, 79-5 Tokiwadai, Hodogaya, Yokohama 240-8501, Japan and Advanced Power Electronics Research Center, National Institute of Advanced Industrial Science and Technology, 1-1-1 Umezono, Tsukuba, Ibaraki 305-8568, Japan but should have been Quantum Information Research Center, Institute of Advanced Sciences, Yokohama National University, 79-5 Tokiwadai, Hodogaya, Yokohama 240-8501, Japan and Department of Physics, Graduate School of Engineering Science, Yokohama National University, 79-5 Tokiwadai, Hodogaya, Yokohama 240-8501, Japan. The original article has been corrected.

