# Supplementary material for: Coherent electric field control of orbital state of a neutral nitrogen-vacancy center
Source: Nat Commun. 2024 May 13;15:4039. doi: 10.1038/s41467-024-47973-3 (PMC11091116; doi:10.1038/s41467-024-47973-3)
Supplement: Supplementary file 1 — Supplementary Information [file 41467_2024_47973_MOESM1_ESM.pdf]

Supplementary Information for: Coherent Electric Field Control of Orbital State of a Neutral Nitrogen-Vacancy Center

Hodaka Kurokawa,[1,*] Keidai Wakamatsu,[2] Shintaro Nakazato,[2] Toshiharu Makino,[3] Hiromitsu Kato,[3] Yuhei Sekiguchi,[1] and Hideo Kosaka[1,2,†]

[1]*Quantum Information Research Center, Institute of Advanced Sciences, Yokohama National University, 79-5 Tokiwadai, Hodogaya, Yokohama 240-8501, Japan*

[2]*Department of Physics, Graduate School of Engineering Science, Yokohama National University, 79-5 Tokiwadai, Hodogaya, Yokohama 240-8501, Japan*

[3]*Advanced Power Electronics Research Center, National Institute of Advanced Industrial Science and Technology, 1-1-1 Umezono, Tsukuba, Ibaraki, 305-8568, Japan*

I. SUPPLEMENTARY NOTES

When the magnetic fields and strain are zero, the Hamiltonian of NV^0 can be written in basis $|\pm\rangle_o = \mp(|e_x\rangle_o \pm i\,|e_y\rangle_o)$ as follows:

$$H_0^{(\pm)}/h = 2\lambda \hat{L}_z \hat{S}_z, \tag{1}$$

where h is the Plank's constant, λ is the spin-orbit interaction parameter, $\hat{L}_z = \sigma_z$, $\hat{L}_\pm = |\pm\rangle_o \langle\mp|_o$ are the orbital operator in the $|\pm\rangle_o = \mp 1/\sqrt{2}(|e_x\rangle_o \pm i\,|e_y\rangle_o)$ basis, $|e_x\rangle_o$ and $|e_y\rangle_o$ are the strain eigenstates, and $S_z = (1/2)\sigma_z$ is the spin operator for the 1/2 spin. Because NV^0 has the same wave function symmetry as the excited state of NV^- due to the dynamic Jahn-Teller effect (the effective C_{3v} symmetry), the effects of the strain and electric fields on $|e_{x,y}\rangle_o$ are considered to be similar to that in the excited state of NV^- [1–3]. Therefore, the discussion on the excited state of NV^- in Ref. [2] is applied on NV^0 as outlined below. In the $|e_{x,y}\rangle_o$ basis, the strain and electric field Hamiltonian can be written as:

$$H_{\text{strain}}^{(e_{x,y})}/h = \epsilon_{A_1}\hat{I} + \epsilon_{E_1}\hat{\sigma}_z - \epsilon_{E_2}\hat{\sigma}_x, \tag{2}$$

$$H_{\text{electric}}^{(e_{x,y})}/h = d_\parallel E_z \hat{I} + d_\perp E_x \hat{\sigma}_z - d_\perp E_y \hat{\sigma}_x, \tag{3}$$

where ϵ_{A_1}, ϵ_{E_1}, and ϵ_{E_2} represent the strain in the energy dimensions with symmetric indices, $d_\perp$ denotes the in-plane electric susceptibility, and $d_\parallel$ represents the electric susceptibility along the NV axis. In the $|\pm\rangle_o$ basis:

$$\begin{aligned} H_{\text{strain}}^{(\pm)}/h = \epsilon_{A_1}\hat{I} - \epsilon_{E_1}(\hat{L}_+ + \hat{L}_-) \\ -\epsilon_{E_2}(-i\hat{L}_+ + i\hat{L}_-), \end{aligned} \tag{4}$$

$$\begin{aligned} H_{\text{electric}}^{(\pm)}/h = d_\parallel E_z \hat{I} - d_\perp E_x(\hat{L}_+ + \hat{L}_-) \\ -d_\perp E_y(-i\hat{L}_+ + i\hat{L}_-). \end{aligned} \tag{5}$$

To simplify this analysis, only the subspace of spin in the state $|\uparrow\rangle_s$ is considered because the strain and electric fields do not directly affect the spin.

* E-mail: kurokawa-hodaka-hm@ynu.ac.jp

† E-mail: kosaka-hideo-yp@ynu.ac.jp

A. Strain and DC electric fields

First, the state where only the strain is present in the system is considered. By redefining the axis of rotation, the strain Hamiltonian can be expressed as follows:

$$H^{(\pm)}_{\text{strain}}/h = \epsilon_{A_1}\hat{I} + \epsilon_{\perp}(\hat{L}_+ + \hat{L}_-). \tag{6}$$

The electric fields are rewritten as shown below because they may not necessarily align in the same direction as the strain:

$$H^{(\pm)}_{\text{electric}}/h = d_{\parallel}E_z\hat{I} + d_{\perp}E_{\perp}(\hat{L}_+ + \hat{L}_-) + d_{\perp}E'_{\perp}(-i\hat{L}_+ + i\hat{L}_-), \tag{7}$$

where $E_{\perp}$ represents the electric fields with the same direction as $\epsilon_{\perp}$, and $E'_{\perp}$ represents the electric fields perpendicular to E_z and $E'_{\perp}$. To diagonalize these equations, $H_0 + H_{\text{strain}} + H_{\text{electric}}$, the energy eigenvalues are obtained as:

$$E_{\pm} = (\epsilon_{A_1} + d_{\parallel}E_z) \pm \sqrt{\lambda^2 + (\epsilon_{\perp} + d_{\perp}E_{\perp})^2 + (d_{\perp}E'_{\perp})^2}. \tag{8}$$

The corresponding eigenvectors without normalization can be written as:

$$\left|+'\right\rangle_{\text{o}} = \frac{\lambda + \sqrt{\lambda^2 + (\epsilon_{\perp} + d_{\perp}E_{\perp})^2 + (d_{\perp}E'_{\perp})^2}}{\epsilon_{\perp} + d_{\perp}E_{\perp} + id_{\perp}E'_{\perp}}\left|+\right\rangle_{\text{o}} + \left|-\right\rangle_{\text{o}}, \tag{9}$$

$$\left|-'\right\rangle_{\text{o}} = \frac{\lambda - \sqrt{\lambda^2 + (\epsilon_{\perp} + d_{\perp}E_{\perp})^2 + (d_{\perp}E'_{\perp})^2}}{\epsilon_{\perp} + d_{\perp}E_{\perp} + id_{\perp}E'_{\perp}}\left|+\right\rangle_{\text{o}} + \left|-\right\rangle_{\text{o}}. \tag{10}$$

B. Strain and AC electric fields

The AC electric fields are applied in such a way that they resonate with the eigenstates of the strain, $\left|\pm'\right\rangle_{\text{o}}$ ($\boldsymbol{E} = 0$). Therefore, the AC electric fields are treated as a perturbation to the eigenstates of the strain.

The effects of the electric fields in the basis of $\left|\pm'\right\rangle_{\text{o}}$ with normalized eigenvectors $\left|+'\right\rangle_{\text{o}} = \alpha\left|+\right\rangle_{\text{o}} + \beta\left|-\right\rangle_{\text{o}}$ and $\left|-'\right\rangle_{\text{o}} = -\beta\left|+\right\rangle_{\text{o}} + \alpha\left|-\right\rangle_{\text{o}}$ can be expressed as:

$$\begin{aligned} H^{(\pm')}_{\text{electric}}/h = d_{\parallel}E_z(t)\hat{I} - d_{\perp}E_x(t)[(\alpha^2 - \beta^2)(\hat{L}_+ + \hat{L}_-) \\ + 2\alpha\beta\hat{L}_z] - d_{\perp}E_y(t)(-i\hat{L}_+ + i\hat{L}_-), \end{aligned} \tag{11}$$

where $\hat{L}_\pm$ are the rising (lowering) operators in the $|\pm'\rangle_{\rm o}$ basis. The equation with the redefined axis after introducing $E''_\perp = \sqrt{(\alpha^2-\beta^2)^2 E_x^2 + E_y^2}$ is :

$$H^{(\pm')}_{\rm electric}/h = d_\parallel E_z(t)\hat{I} + d_\perp E''_\perp(t)(\hat{L}_+ + \hat{L}_-) + 2d_\perp E_x(t)\alpha\beta\hat{L}_z \tag{12}$$

Subsequently, considering the sinusoidal driving fields, we move to the rotating frame with $H_{\rm rot}/h = f_{\rm d}\hat{L}_z/2$ from the system Hamiltonian, $\sqrt{\lambda^2+\epsilon_\perp^2}\hat{L}_z + H^{(\pm')}_{\rm electric}/h$. The equation under the rotating wave approximation is

$$H^{(\pm')}_{\rm electric}/h = \frac{\Delta}{2}\hat{L}_z + \frac{d_\perp E''_\perp}{2}(\hat{L}_+ + \hat{L}_-). \tag{13}$$

Therefore, Rabi oscillation can be observed between the orbital states when $\Delta = 2\sqrt{\lambda^2+\epsilon_\perp^2} - f_{\rm d} \sim 0$.

Also, we briefly mention the derivation of the Autler-Townes splitting in the main text. When we set $\Delta = 0$, $H^{(\pm')}_{\rm electric}/h = d_\perp E''_\perp(\hat{L}_+ + \hat{L}_-)/2$. After the diagonalization of the Hamiltonian, the resultant energy eigenvalues of the dressed states are $\pm d_\perp E''_\perp/2$. The energy splitting between two dressed states is $d_\perp E''_\perp$, corresponding to the Rabi frequency at that driving amplitude. Thus, we can estimate $d_\perp$ from the energy splitting if we know $E''_\perp$.

II. SUPPLEMENTARY METHODS

The electric fields and applied voltage are estimated based on a simulation of the electric field distribution using the finite element method (FEM) (COMSOL Multiphysics, COMSOL). The electric fields at the NV center are estimated to be (E_X, E_Y, E_Z) = (12497.6 V/m, -26122.3 V/m, -7973.57 V/m) per 1 V for the electrode used for DC voltage application, where E_X, E_Y, E_Z are the electric fields in the laboratory frame, as shown in Fig. S1 (a). For the electrode used for ac voltage application, (E_X, E_Y, E_Z) = (13763.6 V/m, -18844.1 V/m, -1079.8 V/m) per 1 V. The electric fields in the NV frame, E_x, E_y, and E_z, are defined as $E_x = E_Y\sin\theta - E_Z\cos\theta$, and $E_y = E_X$, $E_z = -E_Y\cos\theta - E_Z\sin\theta$, where $\sin\theta = \sqrt{1/3}$ and $\sin\theta = \sqrt{2/3}$ (Fig. S1 (b)(c)). Therefore, (E_x, E_y, E_z) = (-8571.3 V/m, 12497.6 V/m, 25932.3 V/m) per 1 V for the electrode used for DC voltage application, and (E_x, E_y, E_z) = (-9998.0 V/m, 13763.6 V/m, 16009.6 V/m) per 1 V for the electrode used for ac voltage application. The applied AC voltage is estimated using the relation $V_{\rm rms} = \sqrt{PR}$,

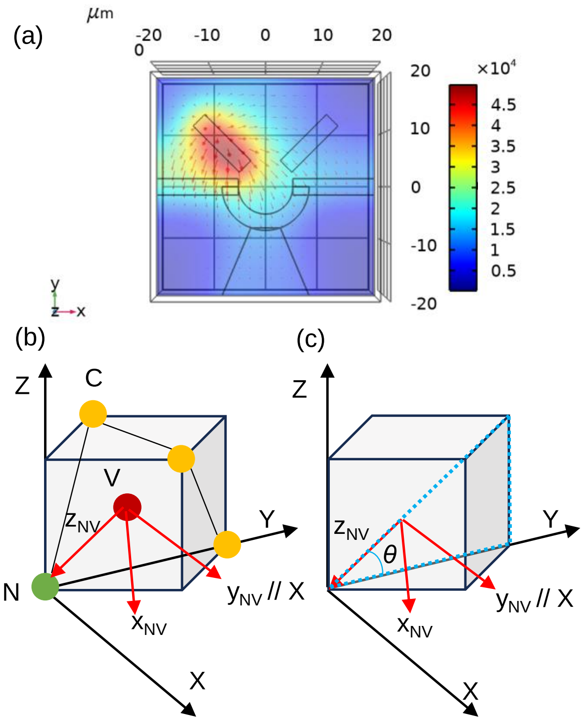

FIG. S1. (a) Electric field distribution around the electrode simulated using FEM software using an applied voltage of 1 V. The unit of the color bar is V/m. (b) Relationship between the laboratory frame and frame of the NV center. C, N, and V correspond to the carbon atom, nitrogen atom, and vacancy, respectively. The direction of the NV center is estimated from the measurement of the optically-detected magnetic resonance. Only two of the four NV axes can be distinguished through measurement; however, the distinction between the two types of axes only affects the sign of the electric field. Therefore, the NV axis is defined as illustrated in the figure. z_{NV} is along the NV axis, and x_{NV} and y_{NV} are defined following the definition of $|e_{x,y}\rangle$ in Ref. [2]. (c) θ is defined as shown in the figure.

where V_{rms} is the root-mean-square (RMS) voltage, P is the microwave power, and R is the resistance. When 1 μW microwave is applied to the electrode at the end of the 50 Ω transmission line, $V_{\mathrm{rms}} = 7.1$ mV. Assuming that the end of the electrode is an approximate open circuit (Z $= \infty$), the voltage amplitude at the electrode is $7.1 \times \sqrt{2} \times 2 = 20$ mV. The factor $\sqrt{2}$ is introduced to convert the RMS voltage to the voltage amplitude, wherein a factor of 2 is used because the circuit is open at the end of the electrode.

III. SUPPLEMENTARY DISCUSSION

In this section, we compare the efficiency of the electric field orbital control with that of the magnetic field spin control to support the argument in the main text that the electric field orbital control can be performed with three orders of magnitude less power than that required for the magnetic field spin control.

If we assume that the NV center is 5 μm away from the electrode, The Rabi frequency for the spin is $\Omega_{\rm s} = g\mu_{\rm B}B_x\hat{S}_x/\hbar$, where g is the electron g-factor, $\mu_{\rm B}$ is the Bohr magneton, B_x is x-axis magnetic field, and $\hat{S}_x$ is the spin operator. When $\Omega_{\rm s}$ is set to be 100 MHz, B_x must be 34.8 (69.7) G for NV^-, spin-1 (NV^0, spin-1/2). From Ampere's law, the required current amplitude is 87.5 (175) mA ($I_{\rm rms} = 87.5(175) \times 1/\sqrt{2}$ mA), corresponding to 191 (765) mW for a 50 Ω transmission line ($P = I_{\rm rms}^2 Z$), where P is power, $I_{\rm rms}$ is the root-mean-square current, Z is impedance. On the other hand, the Rabi frequency for orbital state can be written as $\Omega_{\rm o} = d_\perp E_\perp$, where $d_\perp$ is the electric field susceptibility ($\sim$1000 kHz/V cm^{-1}), and $E_\perp$ is the electric field. When $\Omega_{\rm o}$ is 100 MHz, $E_\perp$=100 V/cm. When NV is 5 μm away from the both electrodes (in between the signal and ground), the required voltage amplitude is 0.1 V ($V_{\rm rms} = 0.1 \times 1/\sqrt{2}$ V), corresponding to 100 μW in the 50 Ω transmission line. Ideally, therefore, power suppression is expected to be more than three orders of magnitude.

In our samples, experimentally, $\sim$500 μW is required to control the orbital state of NV^0 with the Rabi frequency of $\sim$90 MHz, while $\sim$300 mW was needed to control the spin state of NV^- with the Rabi frequency of $\sim$90 MHz. The required power for the magnetic field control is in good agreement with the above estimation. In contrast, the power required for electric field control is larger than our estimation, which may be due to the displacement and misorientation of NV from the ideal position between the electrodes.

---

[1] A. Gali, Physical Review B **79**, 235210 (2009).

[2] J. R. Maze, A. Gali, E. Togan, Y. Chu, A. Trifonov, E. Kaxiras, and M. D. Lukin, New Journal of Physics **13**, 025025 (2011), arXiv:1010.1338.

[3] J. Zhang, C.-Z. Wang, Z. Z. Zhu, and V. V. Dobrovitski, Physical Review B **84**, 035211 (2011).
