# Supplementary material for: Coherent electric field control of orbital state of a neutral nitrogen-vacancy center
Source: Nat Commun. 2024 May 13;15:4039. doi: 10.1038/s41467-024-47973-3 (PMC11091116; doi:10.1038/s41467-024-47973-3)
Supplement: Supplementary file 2 — Peer Review File [file 41467_2024_47973_MOESM2_ESM.pdf]

nature portfolio

Peer Review File

Coherent Electric Field Control of Orbital State of a Neutral Nitrogen-Vacancy Center

Open Access This file is licensed under a Creative Commons Attribution 4.0 International License, which permits use, sharing, adaptation, distribution and reproduction in any medium or format, as long as you give appropriate credit to the original author(s) and the source, provide a link to the Creative Commons license, and indicate if changes were made. In the cases where the authors are anonymous, such as is the case for the reports of anonymous peer reviewers, author attribution should be to 'Anonymous Referee' followed by a clear attribution to the source work. The images or other third party material in this file are included in the article's Creative Commons license, unless indicated otherwise in a credit line to the material. If material is not included in the article's Creative Commons license and your intended use is not permitted by statutory regulation or exceeds the permitted use, you will need to obtain permission directly from the copyright holder. To view a copy of this license, visit http://creativecommons.org/licenses/by/4.0/.

REVIEWER COMMENTS

Reviewer #1 (Remarks to the Author):

The authors demonstrate coherent electrical control of a neutral NV center (NV0) in a diamond crystal. As opposed to the negatively charged NV centers (NV-), there have been fewer studies on the ground state of neutral NV0s. In particular, there has been no demonstration of coherent control directly by microwave driving the transition between the orbital states. Remarkably, the authors report that the electric susceptibility of NV0 in the ground state is similar to that of NV- in the excited state, which paves the way for further studies on quantum device engineering using NV0s. I am thus positive about the manuscript being published, although below are several questions and comments.

- Did the authors measure other NV0s and compare the parameters in the Hamiltonian, such as the spin-orbit interaction \lambda, the strain \epsilon, and overall electric susceptibility?

- In Figure 1 (b), the optical micrograph and PL color map do not look consistent in their shapes. Why?

- I suggest the authors present the temperature dependence of the orbital T1, if ever taken, and discuss what kind of process dominates.

- The data in Figure 3 seems to be taken at zero voltage, right?

Couldn't the broadening also be caused by voltage fluctuation of the measurement line?

- In Figure 5 caption for (b), I would replace \omega by detuning (probably \Delta).

- In the P5 right column in the top paragraph, I disagree with the statement, "the surrounding nuclear spins" as one of the origins of the decoherence. The orbital state must primarily be sensitive to electric field fluctuations, which are not induced by nuclear spins in most circumstances.

- In the P5 right column in the Discussion paragraph, I do not fully understand what "... allowing microwave control using several hundred microwave photons." means. Do the authors want to emphasize an advantage of low-power coherent driving?

- I suggest the papers below also be cited [A. Gali, Phys. Rev. B 79, 235210 (2009)], [P. Siyushev et al., Phys. Rev. Lett. 110, 167402 (2013).]

- In Supplementary Material P2, " BecauseNV0 has the same wave function symmetry as NV-, ..." sounds not entirely correct, as the static Jahn-Teller effect would result in C1h symmetry, according to the paper above by Gali. Perhaps adding "Because of dynamic Jahn-Teller effect" would be better.

Reviewer #2 (Remarks to the Author):

Kurosawa et al present, to my knowledge, the first demonstration of coherent, microwave electric field control of orbital states of a color-center. They utilize the neutral charge state of the NV0 and demonstrate charge and orbital state initialization and coherent control, measuring T1 ~ 137 ns and T2* ~ 30 ns at 5.5 K. They estimate that coherent driving is possible with 3 orders of magnitude lower microwave power due to the stronger electric dipole coupling of the orbital states compared to the spin degree of freedom, making the system promising for experiments in dilution refrigerators with low cooling power. They speculate that the results can be applicable to other widely studied group IV color-centers with similar orbital structures that have lower spectral diffusion and are more promising as spin-photon interfaces than the NV center.

Overall, I believe the work meets the novelty criteria for publication in Nature Communications. However, I am unsure that it meets the criteria for impact, which is my main criticism. I have several questions about the utility of electric field control for quantum information processing that should be clarified before publication.

1. Is there any indication that the orbital degree of freedom will be coherent, even at low temperatures? Since it is so sensitive to electric fields, it is likely to couple to a strong noise bath in the solid state, and this will become even more challenging for example in nanofabricated photonics structures.

2. Is there any strategy or proposal to use this orbital control to encode quantum information on the spin degree of freedom, for example by using a 2-photon transition in an external B field?

3. The authors must more clearly compare this approach with the approach using the strain susceptibility of the orbital states. For example, direct mechanical driving of spin states has been shown for e.g. the GeV center. How does this compare, what are the advantages and disadvantages?

4. It is certainly interesting that this work can be applied to group Iv centers, but the authors need to discuss some of the possible differences, for example the presence of the Jahn Teller effect, and substantially larger spin-orbit couplings (e.g. 50 GHz for SiV- center)

Finally, I have a number of minor questions and comments:

5. The strained eigenstates are introduced in terms of quantities alpha and beta which are never defined or related back to epislon/lambda

6. What is the charge state initialization fidelity?

7. What limits the orbital state initialization fidelity? Why can the authors not use a higher laser power to increase the Fidelity?

8. What are the branching ratios of the optical transitions 2->1 and 2->0? Is single shot orbital readout possible?

9. Note, your T2* is shorter than would be expected from the orbital T1 - can you comment on this?

Reviewer #3 (Remarks to the Author):

The Nitrogen-vacancy (NV) center system has become a versatile experimental platform with many applications ranging from quantum sensing to quantum computation and quantum networking and can be employed both at room and cryogenic temperatures. Surprisingly, besides decades of research on this system, the internal properties of NV centers are still not fully understood. In particular, research on the neutral charge state, NV0, has been lagging.

This article tackles one of these open questions by realizing coherent control of the orbital state in NV0. The authors employ a single NV center to show measurements on the NV0 energy levels, the orbital state's relaxation time, and the optically-detected electric resonance between the two orbital ground states. Further, the established electric field control is used to perform Rabi oscillations between the orbital states and to show a Ramsey measurement.

The paper has a clear story line, is written well and is accessible. It will most likely be of great interest for the community and upcoming experimental works, not only for the NV system, but also for other colour center systems, such as the group-IV centers. The established electric field control might lead to the realization of a hybrid quantum systems, such as an interface for superconducting qubits (microwave-to-optical conversion).

This paper might warrant publication in Nature Comm., however, I have a couple of open points that I would like to see addressed first:

Major comments:

- The authors perform their measurements at ambient magnetic fields at which no Zeeman splitting between the different spin states is observed, i.e. the two spin states remain degenerate. They argue that the spin lifetime (even under laser irradiation) is several milliseconds, long enough as not to influence their measurements. How do the authors justify this claim? While in Ref. [26] such a long lifetime indeed has been shown, in Ref. https://doi.org/10.1038/s41534-022-00637-w a much shorter spin lifetime is observed, either attributed to a higher P1 center concentration or a much lower magnetic field value as within Ref. [26]. Hence, I would argue that one cannot simply claim a certain spin lifetime. Do the authors have experimental data on the actual spin lifetime in their system? Can the authors comment on this major question?

- Error bars: I am missing error bars throughout the whole publication. No error bars are shown on the figures and no error bars are given for experimental fits. I would like to remind the authors that error bars are crucial in our work as experimental scientists and they have to be added for all experimental data and fits.

- One of the main claims within this paper is the much lower level of power required for state manipulation when using orbital states. Can the authors add more information/point to references. At p.5 the authors claim that MW driving of NV- would require three orders of magnitude more power – do the authors have supporting experimental measurements on their claim?

Minor comments:

- P.1; last paragraph: the subscript (s) is introduced even before the spin itself has been introduced

- P.3; first paragraph: The authors extract \lambda = 4.80 GHz from Ref. [26], while there actually a value of 4.9(4)GHz is given. Can the authors comment on this (small) discrepancy?

- P.4; The concept of (a microwave power dependent) Rabi splitting is hardly introduced and it is not easy to follow. Can the authors extend their explanation? In particular I recommend to introduce the concept of Rabi splitting, where it physically comes from and what it can be used for (e.g. to extract certain parameters).

- P.6; For charge initialization the authors employ very strong 637nm resonant pulses (200uW of power). Can the authors comment on the effect of such strong pulses in relation to spectral diffusion? Are such strong pulses needed, or could also lower power charge initialization be employed, as e.g. used in Ref.[26].

We appreciate your careful reviewing of our manuscript. Below is a point-by-point summary of our responses to your comments. Page and line numbers correspond to the numbers in the file indicating the revised differences. We would be happy to respond to your comments and questions.

REVIEWER COMMENTS

Reviewer #1 (Remarks to the Author):

The authors demonstrate coherent electrical control of a neutral NV center (NV^0) in a diamond crystal. As opposed to the negatively charged NV centers (NV^-), there have been fewer studies on the ground state of neutral NV^0 s. In particular, there has been no demonstration of coherent control directly by microwave driving the transition between the orbital states. Remarkably, the authors report that the electric susceptibility of NV^0 in the ground state is similar to that of NV^- in the excited state, which paves the way for further studies on quantum device engineering using NV^0 s. I am thus positive about the manuscript being published, although below are several questions and comments.

(Comment 1-1)

- Did the authors measure other NV^0 s and compare the parameters in the Hamiltonian, such as the spin-orbit interaction ¥lambda, the strain ¥epsilon, and overall electric susceptibility?

(Response 1-1)

We have focused on only one NV presented in the manuscript because other NVs did not show good properties for our experiments as follows. We measured the ground-state splitting of four NVs inside the electrode before we choose the NV presented in the manuscript. Two of them showed large strain splitting of E_x and E_y in the NV^- state, reaching approximately 14 GHz splitting. For these two NVs, we were unable to observe two PLE peaks corresponding to $|0\rangle \rightarrow |2\rangle$ and $|1\rangle \rightarrow |2\rangle$ transitions in NV^0. The other two of them showed relatively small strain splitting of E_x and E_y in the NV^- state, which was around 4 GHz splitting. For these two NVs, we could observe two PLE spectral peaks in the NV^0 state whose ground-state splitting was 13-14 GHz. However, for one of these two, we could not observe any changes in the PLE spectrum induced by dc electric fields. Thus, we chose the remaining NV which was frequency-tunable using dc electric fields and we can access its ground state using our microwave equipment.

(Comment 1-2)

- In Figure 1 (b), the optical micrograph and PL color map do not look consistent in their shapes. Why?

(Response 1-2)

Thank you for pointing out that. In Fig.1 (b) in the manuscript (or Fig. R1 (a) in this response letter), the PL color map looks different from the optical image because of the color bar setting. In Fig.1 (b) in the manuscript, we had to set the saturation intensity to be so low that we could see every NV. Instead, when we set the saturation intensity to be ten times the original one, we can observe the outline of the electrode (Fig. R1 (b)). Also, we can confirm the outline more easily in Fig. R1 (c) with a wider field of view.

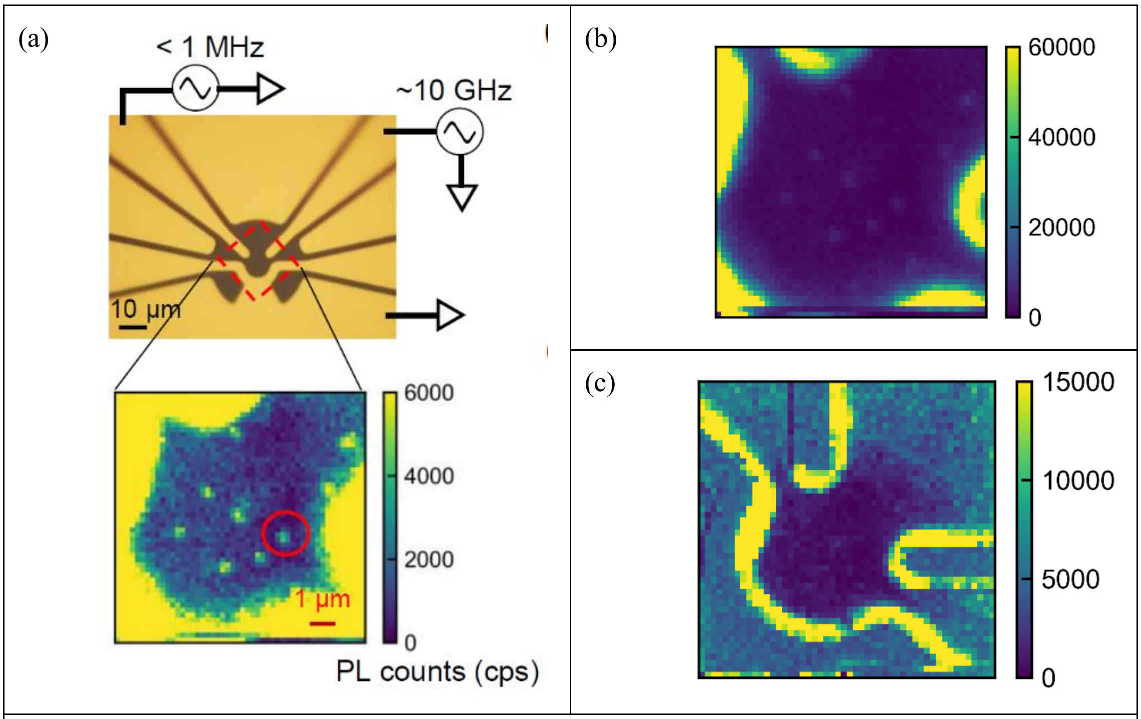

Fig. R1 (a) The optical and PL images of the sample shown in the manuscript. (b) The same data as (a) with different color scale. (c) The PL image with wider field of view.

(Comment 1-3)

- I suggest the authors present the temperature dependence of the orbital T1, if ever taken, and discuss what kind of process dominates.

(Response 1-3)

Thank you for your suggestion. We have not measured the detailed temperature dependence because it has already been evaluated by Baier et al. in Ref. [R1] (Fig. R2). The temperature dependence of the recovery rate, $R_{recovery} = 1/\tau_{recovery} = 1/T_1^{orb}$, is shown in Fig. R2 (b). They attributed the initial linear increase below 9 K to be a single-phonon process. Above 8-9 K, two-phonon Orbach processes were considered to be dominant based on the fitting.

Our T_1^{orb} was shorter than that was reported in Ref. [R1]. We believe that this deviation in T_1^{orb} was caused by the difference of effective temperature at the sample. Due to the thermal radiation from the room temperature objective lens, the local temperature of our sample could be hotter than that of the sample in Ref.[R1].

On the other hand, we are now trying to evaluate temperature dependence of the orbital T_1 in lower temperatures down to 20 mK. Although we have some troubles in cooling down a sample at present, we are looking forward to showing the low temperature measurement results in the near future.

[R1] S. Baier et al., PRL 125, 193601 (2020).

https://journals.aps.org/prl/abstract/10.1103/PhysRevLett.125.193601

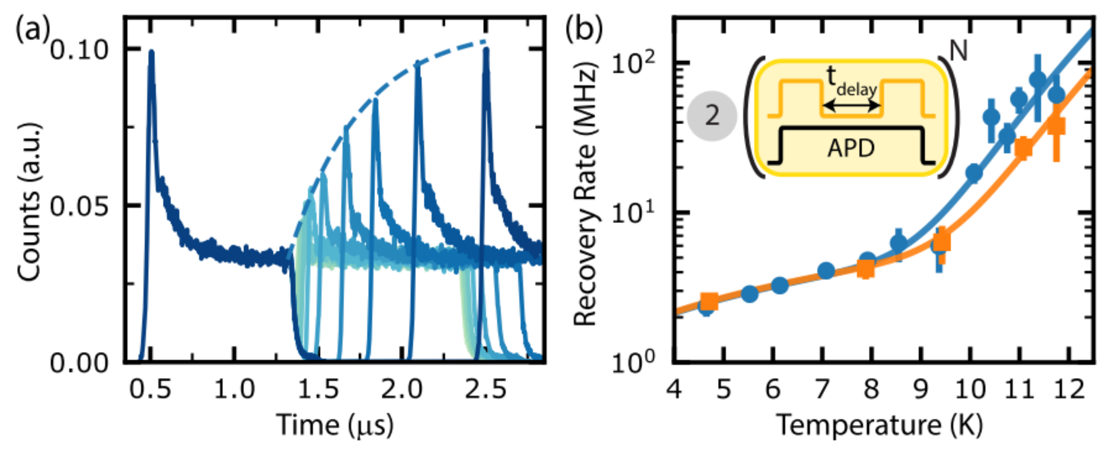

Fig. R2 [**From Ref. [R1], not our data**] (a) The time-dependent fluorescence changing the delay between the first and second pulses. The first pulse corresponds to the orbital initialization and the second pulse is for the readout. (b) The temperature dependence of the orbital recovery rate (~$1/T_1^{orb}$).

(Comment 1-4)

- The data in Figure 3 seems to be taken at zero voltage, right? Couldn't the broadening also be caused by voltage fluctuation of the measurement line?

(Response 1-4)

As you pointed out, the data in Fig. 3 (b) in the manuscript (or Fig. R3 (a) in this response letter) were taken at zero dc voltage with changing frequency of microwave electric fields. Fig. R3 (b) shows the same measurement with termination at the port for the application of dc electric fields. No significant change in FWHM was observed with (FWHM = 130 MHz) and without the termination (FWHM = 145 MHz). This fluctuation in FWHM can be attributed to the error in the fitting or fluctuation in the environment. Thus, it can be said that voltage fluctuation in the dc voltage application line does not have significant impact on this spectral broadening. At present, strong charge initializing 637 nm laser pulse can induce spectral diffusion, limiting the spectral width. Thus, we are now trying to perform experiments at low initialization laser powers by developing a high-quality solid immersion lens with low spectral diffusion and static strain.

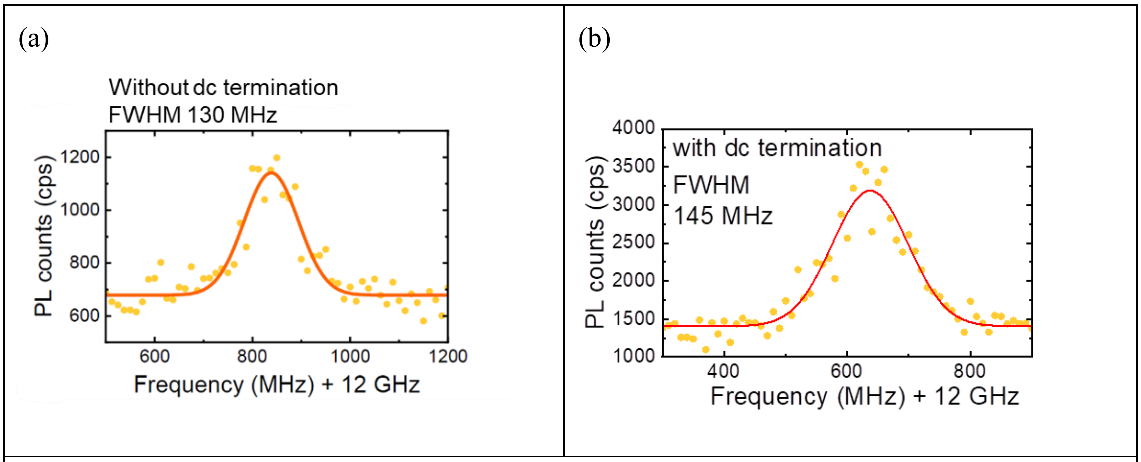

Fig. R3 Optically detected electrical resonance spectrum (a) without and (b) with dc line termination.

(Comment 1-5)

- In Figure 5 caption for (b), I would replace ¥omega by detuning (probably ¥Delta).

(Response 1-5)

Thank you for pointing out that. We changed the sentences as follows.

[before] Figure 5, caption

The blue dots represent the data and the blue curve represents the fitting using the function, $A¥mathrm{sin}(¥omega t+¥phi)¥mathrm{exp}(-t/T_2^*)+B$, where A, B, and $¥phi$ are constants, $¥omega$ is the angular frequency, T_2^* is the orbital coherence time. T_2^* is 30.2 ns.

[after]

The blue dots represent the data and the blue curve represents the fitting using the function, $A\mathrm{sin}(\Delta t+\phi)\mathrm{exp}(-t/T_2^*)+B$, where A, B, and ϕ are constants, Δ is the detuning from the transition between $\ket{0}$ and $\ket{1}$, and T_2^* is the orbital coherence time. T_2^* is 30.2 ns.

(Comment 1-6)

- In the P5 right column in the top paragraph, I disagree with the statement, "the surrounding nuclear spins" as one of the origins of the decoherence. The orbital state must primarily be sensitive to electric field fluctuations, which are not induced by nuclear spins in most circumstances.

(Response 1-6)

Thank you for pointing out that. In the manuscript, we suggested that surroundings spins can cause decoherence because the ground state of NV^0 has both spin and orbital degrees of freedom. However, as you mentioned, the qubit space we focused on has only orbital degree of freedom like $|+\rangle_o|\uparrow\rangle$ and $|-\rangle_o|\uparrow\rangle$. Thus, the main sources for decoherence must be only electric fluctuations and thermal fluctuations (phonons). We changed the sentences as follows:

Page4, line257-

[after]

The origins of the decoherence are attributed ~~to the surrounding nuclear spins ¥cite{Becker2018,Nguyen2019a},~~ spectral diffusion ¥cite{Robledo2010} in the ground state~~,~~ and thermal phonons that are resonant with the orbital transition proposed for SiV ¥cite{Jahnke2015}.

(Comment 1-7)

- In the P5 right column in the Discussion paragraph, I do not fully understand what "... allowing microwave control using several hundred microwave photons." means. Do the authors want to emphasize an advantage of low-power coherent driving?

(Response 1-7)

Yes, we wanted to emphasize an advantage of low-power coherent driving in these sentences. We added words and sentences to emphasize the advantages and future perspectives as shown below.

[after] Page5, line279-

If a high impedance superconducting microwave resonator with a zero-point voltage fluctuation of tens of microvolts ¥cite{Samkharadze2016,Niepce2019} is integrated, the single-photon coupling is expected to be tens of kilohertz, allowing further low-power microwave control using several hundred microwave photons. If we can further increase the coupling to $¥sim$1 MHz by bringing the electrode and the color center closer together, we can reach the strong coupling regime of the color center and the microwave resonator, where we can observe single-photon interaction.

(Comment 1-8)

- I suggest the papers below also be cited [A. Gali, Phys. Rev. B 79, 235210 (2009)], [P. Siyushev et al., Phys. Rev. Lett. 110, 167402 (2013).]

(Response 1-8)

Thank you for your suggestion. We added a citation as follows (Refs. [22] and [25]).

[after]

[illegible]

[22] A. Gali, Physical Review B **79**, 235210 (2009).

[23] M. V. Hauf, B. Grotz, B. Naydenov, M. Dankerl, S. Pezzagna, J. Meijer, F. Jelezko, J. Wrachtrup, M. Stutzmann, F. Reinhard, and J. A. Garrido, Physical Review B **83**, 081304 (2011).

[24] B. Grotz, M. V. Hauf, M. Dankerl, B. Naydenov, S. Pezzagna, J. Meijer, F. Jelezko, J. Wrachtrup, M. Stutzmann, F. Reinhard, and J. A. Garrido, Nature Communications **3**, 729 (2012).

[25] P. Siyushev, H. Pinto, M. Vörös, A. Gali, F. Jelezko, and J. Wrachtrup, Physical Review Letters **110**, 167402 (2013).

[26] Y. Doi, T. Makino, H. Kato, D. Takeuchi, M. Ogura, H. Okushi, H. Morishita, T. Tashima, S. Miwa, S. Ya-

(Comment 1-9)

- In Supplementary Material P2, " BecauseNV0 has the same wave function symmetry as NV-, ..." sounds not entirely correct, as the static Jahn-Teller effect would result in C1h symmetry, according to the paper above by Gali. Perhaps adding "Because of dynamic Jahn-Teller effect" would be better.

(Response 1-9)

Thank you for your suggestion. We changed sentences as follows:

[before]

Because NV^0 has the same wave function symmetry as NV$^-$, the effects of the strain and electric fields on $¥ket{e_{x,y}}_{¥text{o}}$ are considered to be similar to that in the excited state of NV$^-$ ¥cite{Maze2011}.

[after] Page2, line 20- Supplementary materials

Because NV^0 has the same wave function symmetry as the excited state of NV$^-$ due to the dynamic Jahn-Teller effect (the effective C_{3v} symmetry), the effects of the strain and electric fields on $¥ket{e_{x,y}}_{¥text{o}}$ are considered to be similar to that in the excited state of NV$^-$ ¥cite{ Gali2009,Maze2011,Zhang2011}.

[2] J. R. Maze, A. Gali, E. Togan, Y. Chu, A. Trifonov, E. Kaxiras, and M. D. Lukin, New Journal of Physics 13, 025025 (2011), arXiv:1010.1338.

[3] J. Zhang, C.-Z. Wang, Z. Z. Zhu, and V. V. Dobrovitski, Physical Review B 84, 035211 (2011).

Reviewer #2 (Remarks to the Author):

Kurosawa et al present, to my knowledge, the first demonstration of coherent, microwave electric field control of orbital states of a color-center. They utilize the neutral charge state of the NV0 and demonstrate charge and orbital state initialization and coherent control, measuring T1 ~ 137 ns and T2* ~ 30 ns at 5.5 K. They estimate that coherent driving is possible with 3 orders of magnitude lower microwave power due to the stronger electric dipole coupling of the orbital states compared to the spin degree of freedom, making the system promising for experiments in dilution refrigerators with low cooling power. They speculate that the results can be applicable to other widely studied group IV color-centers with similar orbital structures that have lower spectral diffusion and are more promising as spin-photon interfaces than the NV center.

Overall, I believe the work meets the novelty criteria for publication in Nature Communications. However, I am unsure that it meets the criteria for impact, which is my main criticism. I have several questions about the utility of electric field control for quantum information processing that should be clarified before publication.

(Comment 2-1)

1. **Is there any indication that the orbital degree of freedom will be coherent, even at low temperatures? Since it is so sensitive to electric fields, it is likely to couple to a strong noise bath in the solid state, and this will become even more challenging for example in nanofabricated photonics structures.**

(Response 2-1)

As we presented in the manuscript, the orbital coherence time, T_2*, was 20-30 ns at ~5 K. At this temperature, T_2* is limited by the charge (electric field) fluctuations and thermal noise (phonons) around ~13 GHz. Our recent experiments indicate that the spectral diffusion during the charge initialization mainly limits the T_2* (See Response 2-9 in detail). Thus, we expect the elongation of T_2* by suppressing the spectral diffusion using a high-quality sample with smaller charge fluctuations. Furthermore, if we further decrease temperature down to tens of millikelvins, thermal noise can be suppressed dramatically as was observed in group IV vacancy centers. Thus, only the charge fluctuations would limit the coherence time at tens of millikelvins. We expect the extension of T_2* (or T_2^{echo}) at tens of millikelvin and are now trying to begin low temperature measurements using a dilution refrigerator.

In addition, the spectral diffusion (or charge fluctuations) in nanostructures is the biggest problem that must be addressed. We have two expectations to suppress these charge fluctuations. First, the improvement of fabrication techniques can suppress the charge fluctuations. We are now trying to develop photonic crystal structures using ICP etching with our collaborators. We have observed narrow optical linewidth in NV^-, down to 30 MHz using only a resonant 637 nm laser after the vertical etching of nanophotonic structures. Although the structure is not a fully suspended photonic crystal, the result is promising for us. Besides, in Ref. [R2], a lifetime-limited linewidth was observed in a single-scan experiment of NV centers in diamond nanopillars. Thus, we have a hope that we can suppress charge fluctuations by improving the fabrication process even in NV centers.

Second, if we can fabricate a photonic nanostructure with extremely high quality (Cooperativity~100 [R3]), we can read out the qubit state using only a single photon. In such a situation, excess photon absorption in a diamond, which causes charge fluctuations, can be suppressed.

[R2] L. Orphal-Kobin et al., PRX 13, 011042 (2023).
https://journals.aps.org/prx/abstract/10.1103/PhysRevX.13.011042
[R3] M. Bhaskar et al., Nature 580, 7801 (2020).
http://www.nature.com/articles/s41586-020-2103-5

(Comment 2-2)

2. Is there any strategy or proposal to use this orbital control to encode quantum information on the spin degree of freedom, for example by using a 2-photon transition in an external B field?

(Response 2-2)

Yes, as you pointed out, it is possible to transfer the orbital state to the spin state using the two-photon Raman transition under an external ***B*** field. Fig. R4 shows an example. The microwave electric field is applied to drive $|-\rangle_0|\uparrow\rangle_S$ to $|+\rangle_0|\uparrow\rangle_S$. The Rabi frequency of this transition is Ω_1 and the detuning is Δ. At the same time, the microwave magnetic field is applied to drive $|+\rangle_0|\uparrow\rangle_S$. to $|+\rangle_0|\downarrow\rangle_S$. The Rabi frequency of this transition is Ω_2, and the detuning, Δ, is equal to the driving electric field. Then, the resulting two-photon Rabi frequency, Ω_{raman}, becomes $\Omega_1\Omega_2/2\Delta$. When we set $\Omega_1/2\pi = 100$ MHz, $\Omega_2/2\pi =$ 10-100 MHz, $\Delta/2\pi = 500$ MHz, $\Omega_{\text{raman}}/2\pi = 1 - 10$ MHz.

Since the orbital lifetime, T_1^{orb}, is limited to several hundred of nanoseconds at most at 5 K, the operating speed defined by Ω_{raman} is not sufficient to perform the transfer within T_1^{orb}. However, as a preliminary result, we observed an increase of T_1^{orb} up to ~4 us below 300 mK. Thus, we can test the effectiveness of this scheme using a dilution refrigerator.

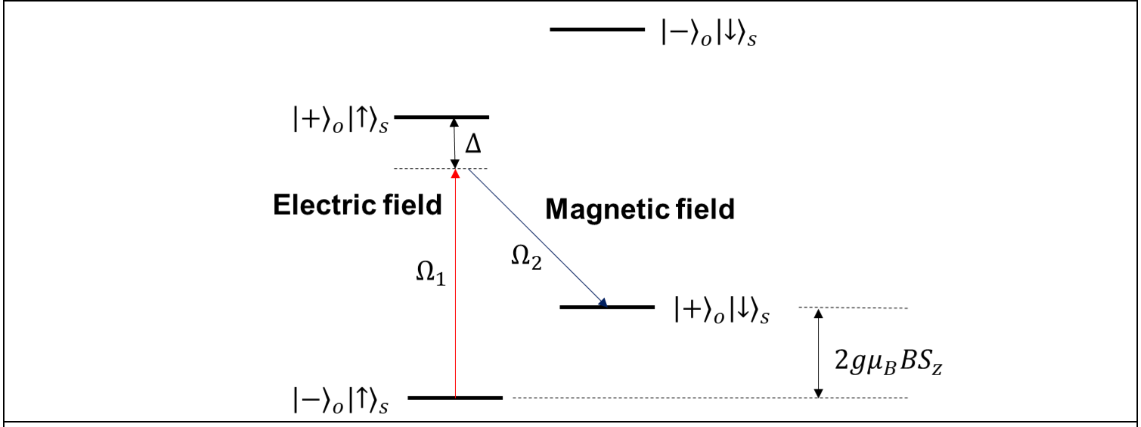

Fig. R4 The ground state energy diagram of NV^0 under external magnetic field. The electric field and magnetic field with detuning are applied to induce two-photon transition.

(Comment 2-3)

3. **The authors must more clearly compare this approach with the approach using the strain susceptibility of the orbital states. For example, direct mechanical driving of spin states has been shown for e.g. the GeV center. How does this compare, what are the advantages and disadvantages?**

(Response 2-3)

Thank you for your suggestion. Compared to the typical mechanical driving, our electric field control has two main advantages. The first advantage is frequency tunability. Typical GHz mechanical driving devices use mechanical resonators like surface acoustic wave resonator (SAW) and bulk acoustic wave resonator (BAW). It is difficult to tune their resonance frequency even in the small frequency range (~MHz) in these kinds of resonators. On the other hand, electric fields can be applied using simple electrodes and the applicable microwave frequency is not limited by its structure. Furthermore, even if we use a microwave (electromagnetic field) resonator to increase the interaction between NV^0 and electric fields, the resonance frequency of the microwave resonator can be tuned from several hundred of MHz to GHz depending on the design of the microwave resonator.

The second advantage is the easiness in fabrication. To excite a mechanical wave in a diamond, deposition of piezoelectric material is necessary. Also, an interdigital transducer with several hundred nanometers must be fabricated to excite a surface acoustic wave. The bulk acoustic wave device also needs more processes (fabrication of top and bottom electrodes and piezoelectric material in between) compared to the simple fabrication of the electrode.

The disadvantages are as follows. First, high electric field susceptibility of NV^0 can be more easily affected by charge fluctuations compared to group IV color centers, resulting in spectral instability (diffusion). Thus, we need to pay much more effort to suppress charge fluctuations compared to group IV color centers if we need a stable spectrum.

We added this comparison to the main text as follows:

[before]

Moreover, controlling the orbital state using phonons is promising. Since the mode volume of the phononic resonator can be designed to be several orders of magnitude smaller than that of typical microwave resonators ¥cite{Neuman2021,Kim2023}, the single-phonon coupling can exceed 1 MHz, allowing the realization of a strong coupling between the single phonon and NV^0.

[after] Page5, line 289-

Also, we should compare the electric field control of the orbital state with another driving scheme, mechanical driving ¥cite{Maity2020}. The electric field driving requires less complex devices and offers wide frequency range while typical mechanical oscillators employed in experiments are mechanical resonators with narrow frequency range around resonant frequency ¥cite{Chen2018a,Maity2020}. However, though device fabrication processes and frequency tuning result in some more complexity in experiments, controlling the orbital state using phonons is still promising. Since the mode volume of the phononic resonator can be designed to be several orders of magnitude smaller than that of typical microwave resonators ¥cite{Neuman2021,Kim2023}, the single-phonon coupling can exceed 1 MHz without the use of extremely close proximity electrodes as mentioned above, allowing to reach the strong coupling regime with maintaining both microwave and optical access.

(Comment 2-4)

4. **It is certainly interesting that this work can be applied to group Iv centers, but the authors need to discuss some of the possible differences, for example the presence of the Jahn Teller effect, and substantially larger spin-orbit couplings (e.g. 50 GHz for SiV- center)**

(Response 2-4)

Thank you for pointing out those things. Indeed, the Jahn Teller effect have an impact on Hamiltonian of NV center and group IV color centers. According to Refs. [R4-R6], those color centers can be described in the same vibronic model, $E \otimes e$, where E denotes orbitally doubly degenerated electronic state and e denotes a doubly degenerate vibrational mode. When the system can be described using the same model, the effect of the Jahn Teller effect on the Hamiltonian can be written in the same way. Thus, the same effect on the energy can be expected qualitatively. However, the coefficients of I, σ_x, σ_z in the Hamiltonian could be different in these color centers. To compare these values in detail, DFT calculations on each color center is required as was performed for the excited state of NV^- [R6].

Also, the difference in spin-orbit coupling parameter leads quantitative difference in relevant energy scale (not qualitative difference). For NV^0 with ~10 GHz spin-orbit splitting, the strain exceeding ~20 GHz in energy can change its eigenstate from $|\pm\rangle_o$ to $|+\rangle_o \pm |-\rangle_o$. On the other hand, to control its eigenstate from no-strain limit to highly strained limit, much larger strain is required for SiV or other group IV color centers.

Another difference between NV^0 and group IV color center is in their optical excited state. While NV^0 has two optically excited states with two spin degrees of freedom and one orbital degree of freedom, group IV color centers have for optically excited states with two spin degrees of freedom and two orbital degrees of freedom.

We added this comparison to the main text as follows:

[after] Page 6, line308-

In addition, a comparison with group IV color centers needs to be discussed. Although the ground state can be modeled with the same Hamiltonian for NV^0 and group IV color centers, considering the spin-orbit coupling, the spin (orbital) Zeeman effect, the strain, and the Jahn-Teller effect which is indistinguishable from strain, the coefficients in each term are different. The largest difference in parameters would be the spin-orbit coupling, which ranges from $¥sim$ 5 GHz in NV^0 to $¥sim$ 400 GHz (corresponding to the splitting of $¥sim$ 800 GHz) in PbV. The ratio of spin-orbit coupling to strain determines the degree of

hybridization of the ground state wavefunction, which is essential for magnetic field control of the ground state spin. For NV^0 with 5 GHz spin-orbit coupling, several GHz to tens of GHz of strain parameters are sufficient to fully tune its wavefunction from the spin-orbit coupling dominant regime to the strain dominant regime. In contrast, tens to hundreds of GHz of strain are required to fully control the wavefunction of group IV color centers. Furthermore, we can control the Hamiltonian of NV^0 using dc electric fields, effectively suppressing or enhancing the effect of static strain. Thus, NV^0 can be a good platform to study the effect of strain on the Hamiltonian in a wide parameter range with relatively small strain parameters and dc electric fields. It should be noted that the excited state Hamiltonian of NV^0 and group IV color centers are different. While NV^0 has two optically excited states with two spin degrees of freedom and one orbital degree of freedom, group IV color centers have optically excited states with two spin degrees of freedom and two orbital degrees of freedom. This difference may affect the readout scheme when considering the single-photon readout demonstrated in Ref. ¥cite{Nguyen2019}.

[R4] C. Hepp et al., PRL 112, 036405 (2014). C. Hepp, PhD thesis.
https://journals.aps.org/prl/abstract/10.1103/PhysRevLett.112.036405
[R5] J. Zhang et al., PRB 97, 165204 (2018).
https://journals.aps.org/prb/abstract/10.1103/PhysRevB.97.165204
[R6] T. A. Abtew et al., PRL 107, 146403 (2011).
https://journals.aps.org/prl/abstract/10.1103/PhysRevLett.107.146403

(Comment 2-5)

Finally, I have a number of minor questions and comments:

5. **The strained eigenstates are introduced in terms of quantities alpha and beta which are never defined or related back to epislon/lambda**

(Response 2-5)

Sorry for not mentioning it enough. We wrote down the strained eigenstates using epsilon/lambda in eq. (9)(10) in the Supplementary materials though it is unnormalized form. We add mention in the manuscript to refer the Supplementary materials.

[after] Page1, line80-

Under strain, the eigenstates change from $¥ket{¥pm}_{¥text{o}}$ to $¥ket{+'}_{¥text{o}}=¥alpha¥ket{+}_{¥text{o}}+¥beta¥ket{-}_{¥text{o}}$ and $¥ket{-'}_{¥text{o}}=-¥beta¥ket{+}_{¥text{o}}+¥alpha¥ket{-}_{¥text{o}}$ with $¥alpha^2+¥beta^2=1$ (See Supplementaly eq. (9)(10)).

(Comment2-6)

6. **What is the charge state initialization fidelity?**

(Response 2-6)

The charge state initialization fidelity slightly depended on the initialization power. Here, we estimate it from the residual offset count after the irradiation of long charge initialization pulse of 637 nm. The time-dependent fluorescence are shown in Fig. R5. Fig. R5 (a) and (b) correspond to 10 uW and 200 uW initialization power, respectively. From the exponential fit, the residual offset ranged from 2.0-3.0% (fidelity : 97.0-98.0%) at 10 uW (power of 637 nm laser) to 0.1-3.0% (fidelity : 97.0-99.9 %) at 200 uW. The uncertainty comes from the uncertainty of the background counts. We added the following sentence in the Methods section.

[after] Page6 line 389

The charge initialization fidelity is estimated to be $>97.0¥%$ from the residual fluorescence after the irradiation of long charge initialization pulse.

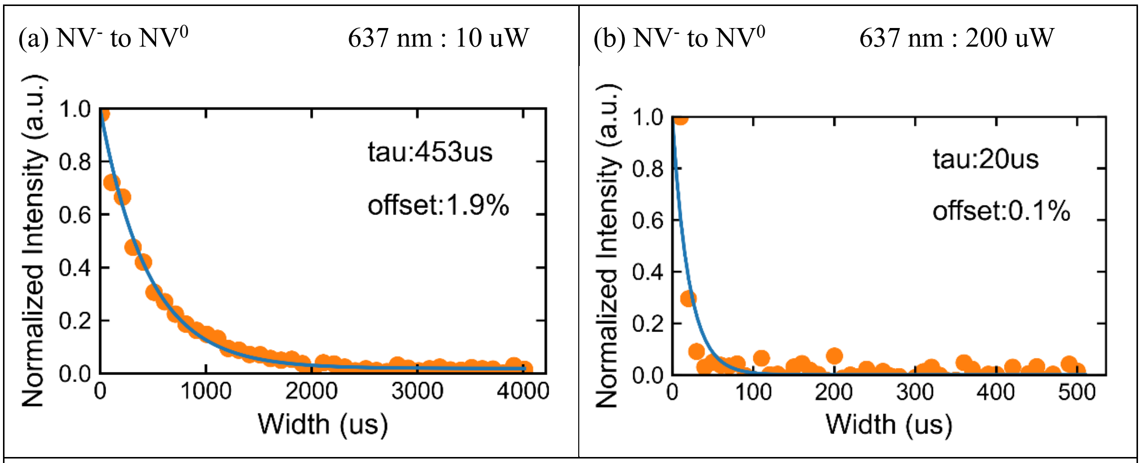

Fig. R5 The decay of the fluorescence from NV^- depending on the width of 637 nm resonant laser. (a) The power of the 637 nm laser is 10 uW. (b) The power of the 637 nm laser is 200 uW.

(Comment 2-7)

7. What limits the orbital state initialization fidelity? Why can the authors not use a higher laser power to increase the Fidelity?

(Response 2-7)

Both the orbital relaxation time and the laser power determine the orbital state initialization fidelity. In our experiments, the initialization laser power did not limit the initialization fidelity as shown in Fig. R6 (a). When we changed the yellow (575 nm) laser power from 200 nW to 3000 nW, remarkable changes in the intensity (ratio) were not observed.

On the contrary, the orbital relaxation time seems to limit the fidelity dominantly. As a preliminary result, Fig. R6 (b) shows the time-dependent fluorescence measured at 15 mK. The decrease of the residual fluorescence can be clearly seen compared to Fig. R6 (a) taken at 4 K. At this temperature, the orbital relaxation time reached 1.5-2.0 us. This increase in orbital relaxation time contributed to the increase in the initialization fidelity (reduction of the residual fluorescence). We are now trying to study whether or not complete (near 100%) orbital initialization is possible.

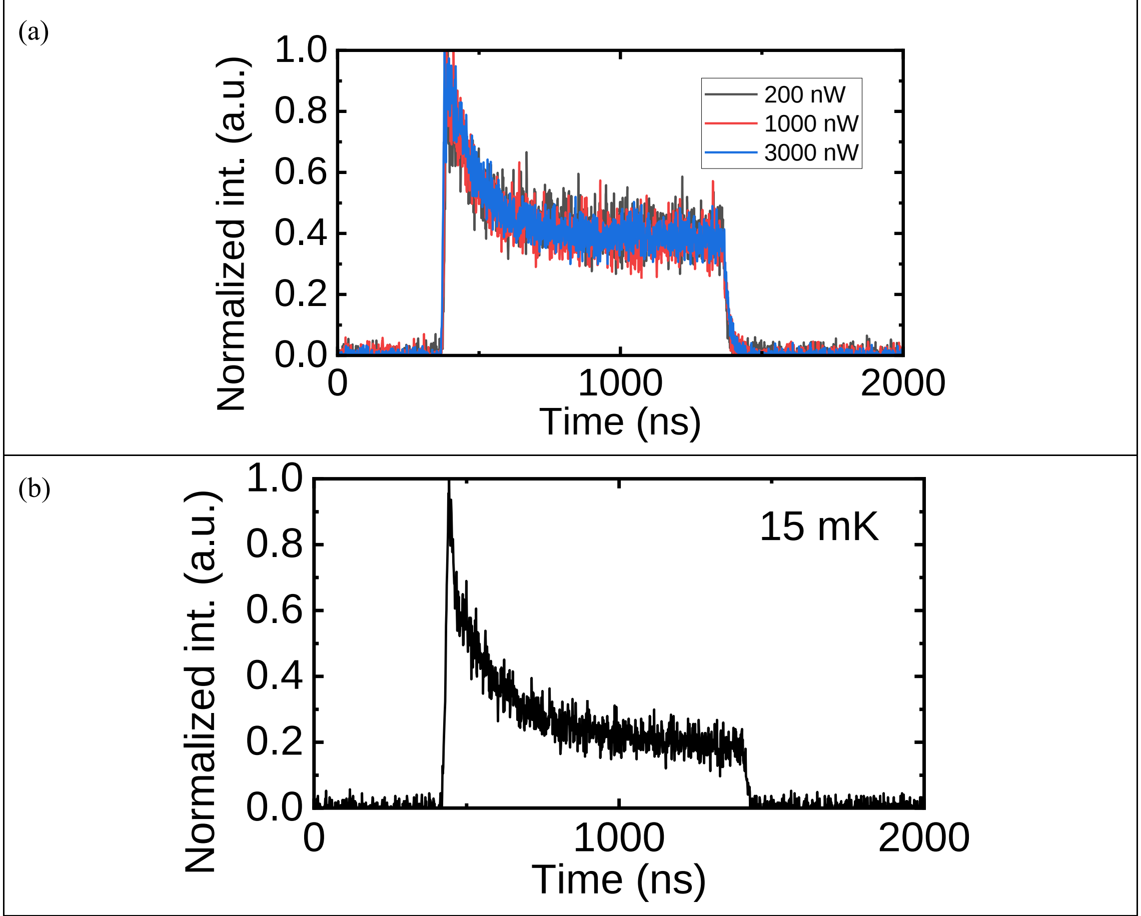

Fig. R6 Time-dependent fluorescence from NV^0 under the resonant 575 nm excitation. (a) The measurement at 4 K changing the 575nm laser power. (b) The measurement at 15 mK.

(Comment 2-8)

8. **What are the branching ratios of the optical transitions 2->1 and 2->0?**

(Response 2-8)

In the manuscript, we defined $|0\rangle, |1\rangle, |2\rangle$ as the subspace of the ground state of NV^0 with same spin. The transition from 2 ($|0\rangle_o|\downarrow\rangle_s$)->1 ($|-\rangle_o|\downarrow\rangle_s$) and 2 ($|0\rangle_o\big||\downarrow\rangle\big\rangle_s$)->0($|-\rangle_o|\downarrow\rangle_s$) or 2 ($|0\rangle_o|\uparrow\rangle_s$)->1 ($|+\rangle_o|\uparrow\rangle_s$) and 2 ($|0\rangle_o|\uparrow\rangle_s$)->0($|-\rangle_o|\uparrow\rangle_s$) do not involve spin-flipping process. Though we do not have experimental evidence, it is plausible that $|0\rangle_o|\downarrow\rangle_s$)->$|-\rangle_o|\downarrow\rangle_s$ and $|0\rangle_o|\downarrow\rangle_s$)->$|+\rangle_o|\downarrow\rangle_s$ show same transition probability considering the difference of L_z in both transitions are ± 1.

9. Is single shot orbital readout possible?

(Response2-9)

Since the orbital T_1 is short (1-2 us @ 15 mK) in our current sample and technology, it is difficult to perform a single-shot readout of the orbital state in the usual manner. However, theoretically, single-shot readout could be possible via orbital-to-spin conversion as shown in Fig. R4. Fig. R7 (a)(b) shows an idea for orbital-to-spin conversion via two-photon Raman transition. Depending on the initial orbital state, the spin of the resultant state becomes different. Since single-shot readout of the spin state has already been performed in Ref. [R1], we can distinguish the initial orbital state in a single measurement by reading the spin state.

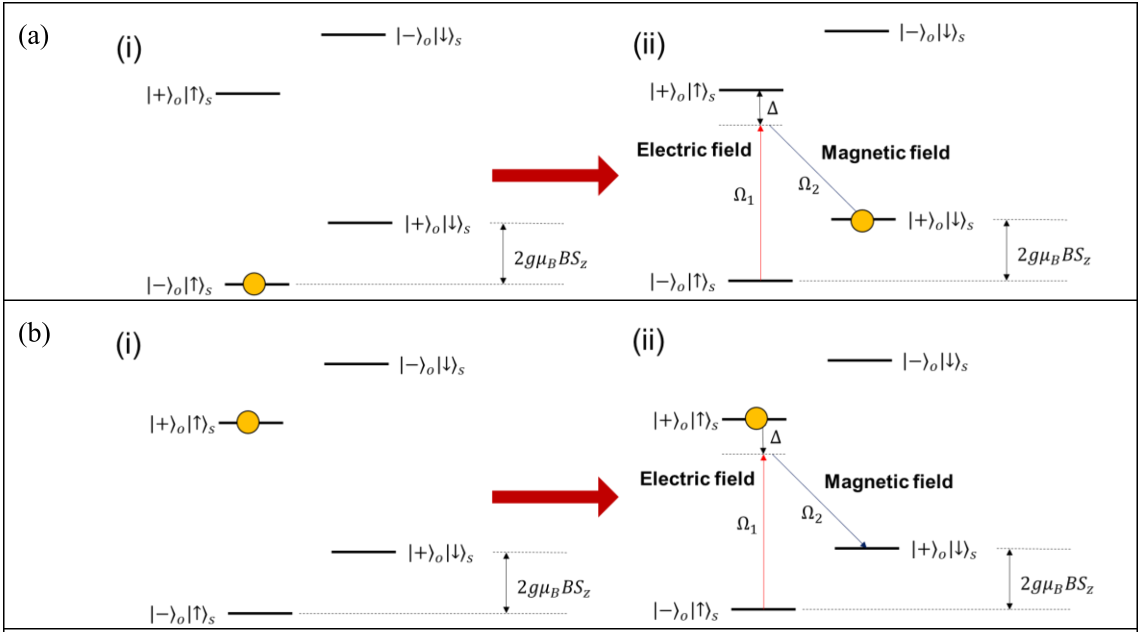

Fig. R7 One scheme to perform single-shot readout of the orbital state. (a) When the state is in $|-\rangle_o|\uparrow\rangle_s$, it is transferred to $|+\rangle_o|\downarrow\rangle_s$ via two-photon Raman transition. (b) When the state is in $|+\rangle_o|\uparrow\rangle_s$, it remains the same state via two-photon Raman transition. As a result, different orbital states map to different spin states. Since the single-shot readout of the spin states have already demonstrated, we can distinguish the initial orbital state by reading the resultant spin state using single-shot spin readout technique.

[R1] S. Baier et al., PRL 125, 193601 (2020).
https://journals.aps.org/prl/abstract/10.1103/PhysRevLett.125.193601

(Comment 2-9)

10. Note, your T2* is shorter than would be expected from the orbital T1 - can you comment on this?

(Response 2-9)

Thank you for your comment on that. We are now trying to understand the limitation in T_1 and T_2(*). Although it is still a preliminary result, we recently observed an approximately 10-fold (or even more) increase in T_2 using the Hahn-echo technique. Based on this result, we have a hypothesis that spectral diffusion can have a dominant effect in the decrease of T_2^*. In our experiments, we repeat the same laser pulse sequence 10^7-10^8 times to obtain one point in the Rabi (Ramsey) oscillations. During this long repetition, the spectral diffusion must occur. If spectral diffusion occurs randomly in the initialization and readout process, we can model the Ramsey interference as the summation of the ensemble of cosine curves with slightly different detunings, Δ. As a first approximation, we do not introduce T_2* decay in the summation.

Fig. R8 shows the summation $\cos(\Delta t)$ 10^6 times. Here, we have assumed that Δ follows a normal distribution with a mean of 58 MHz and a variance of 7 MHz. Even if we do not introduce T_2* into the calculation, we can reproduce the decay in the ensemble averaged intensity, which is similar to the decay curve observed in our experiments. Thus, we want to measure the intrinsic T_2* in the sample with small spectral diffusion or perform a measurement after the charge-resonance check.

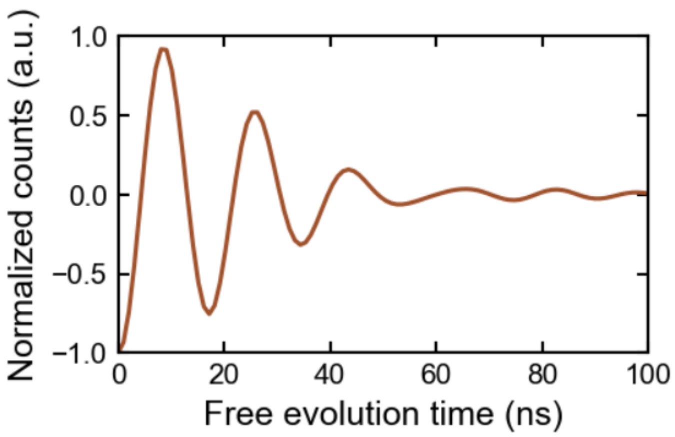

Fig. R8 10^6 ensemble average of $\cos(\Delta t)$, whereΔ follows a normal distribution with center 58 MHz and variance 7 MHz.

Reviewer #3 (Remarks to the Author):

The Nitrogen-vacancy (NV) center system has become a versatile experimental platform with many applications ranging from quantum sensing to quantum computation and quantum networking and can be employed both at room and cryogenic temperatures. Surprisingly, besides decades of research on this system, the internal properties of NV centers are still not fully understood. In particular, research on the neutral charge state, NV0, has been lagging.
This article tackles one of these open questions by realizing coherent control of the orbital state in NV0. The authors employ a single NV center to show measurements on the NV0 energy levels, the orbital state's relaxation time, and the optically-detected electric resonance
between the two orbital ground states. Further, the established electric field control is used to perform Rabi oscillations between the orbital states and to show a Ramsey measurement.
The paper has a clear story line, is written well and is accessible. It will most likely be of great interest for the community and upcoming experimental works, not only for the NV system, but also for other colour center systems, such as the group-IV centers. The established electric field control might lead to the realization of a hybrid quantum systems, such as an interface for superconducting qubits (microwave-to-optical conversion).
This paper might warrant publication in Nature Comm., however, I have a couple of open points that I would like to see addressed first:

Major comments:

(Comment 3-1)

- The authors perform their measurements at ambient magnetic fields at which no Zeeman splitting between the different spin states is observed, i.e. the two spin states remain degenerate. They argue that the spin lifetime (even under laser irradiation) is several milliseconds, long enough as not to influence their measurements. How do the authors justify this claim? While in Ref. [26] such a long lifetime indeed has been shown, in Ref. https://doi.org/10.1038/s41534-022-00637-w <https://doi.org/10.1038/s41534-022-00637-w> a much shorter spin lifetime is observed, either attributed to a higher P1 center concentration or a much lower magnetic field value as within Ref. [26]. Hence, I would argue that one cannot simply claim a certain spin lifetime. Do the authors have experimental data on the actual spin lifetime in their system? Can the authors comment on this major question?

(Response 3-1)

Thank you for your pointing that. We noticed Ref. [R7] and we agree with your comment that we cannot insist on the (long) spin lifetime without experimental evidence. It is difficult to directly measure the spin lifetime at zero magnetic field under our current experimental setup. However, we consider that Ref. [R7] does not affect our results and interpretations seriously.

In Ref [R1](=Ref. [26]), the spin lifetime in the NV^0 state ranged from 90 ms (under resonant laser irradiation) to 1.5 s (without laser), while it was 570 us (including NV^0 to NV^- switching). Our typical experimental timescale was ~3 us (including repetition cycle with orbital initialization : 200-300 us), which was even shorter than the both 90 ms and 570 us. In addition, even if the spin flip occurs a few times during the repetition, microwave electric fields can drive both spin states, inducing the Rabi oscillation. Also, we assumed that we set the polarization of 575 nm readout laser to be spin-independent. Thus, we changed the main text as follows:

[before]

~~This assumption holds as long as the laser irradiation time is sufficiently short because the lifetime of the spin reaches several milliseconds even under laser irradiation ¥cite{Baier2020}.~~

[after] Page2, line105-

This assumption holds for spin-independent experiments or experiments with time scales shorter than spin relaxation time. Basically, our experiments are assumed to be spin-independent because we can drive and readout both spin states optically and electrically. Furthermore, although reported spin relaxation times ranges from 570 $¥mu$s to 1.5 s

depending on situations ¥cite{Baier2020, Bradley2022}, they are still longer than the timescale of our experiments ($¥sim$ a few $¥mu$s).

[R1] S. Baier et al., PRL 125, 193601 (2020).
https://journals.aps.org/prl/abstract/10.1103/PhysRevLett.125.193601
[R7] C. Bradley et al., npj Quantum Information 8, 122 (2022).

(Comment 3-2)

- Error bars: I am missing error bars throughout the whole publication. No error bars are shown on the figures and no error bars are given for experimental fits. I would like to remind the authors that error bars are crucial in our work as experimental scientists and they have to be added for all experimental data and fits.

(Response 3-2)

Thank you for your guidance. As you pointed out, error bars are indeed very important. We added error bars corresponding to the standard deviation in each graph as possible. Also, errors in fitting parameter are now also shown (also the standard deviation).

(Comment 3-3)

- One of the main claims within this paper is the much lower level of power required for state manipulation when using orbital states. Can the authors add more information/point to references. At p.5 the authors claim that MW driving of NV- would require three orders of magnitude more power – do the authors have supporting experimental measurements on their claim?

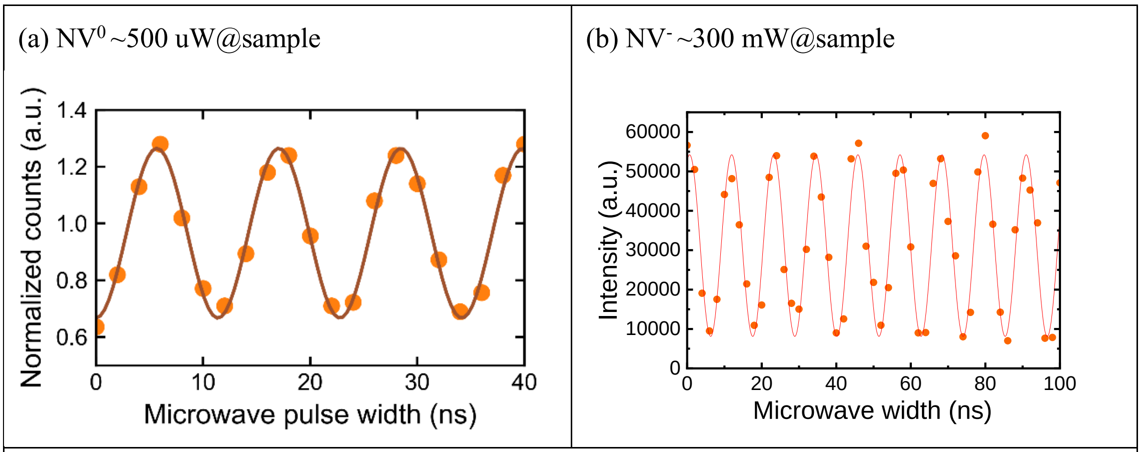

Fig. R9 (a) The orbital state Rabi oscillation in NV^0. (b) The spin Rabi oscillation in NV^-. Those measurements are performed in different samples with the same electrode configuration.

(Response 3-3)

Thank you for your pointing out that. We estimated the value as follows. Here, we assume the NV center 5 um away from the electrode. The Rabi frequency for spin is $\Omega_s = g\mu_B B_x S_x/\hbar$, where g is the electron g-factor, μ_B is the Bohr magneton, B_x is x-axis magnetic field, and S_x is the spin operator. When Ω_s is set to be 100 MHz, B_x must be 34.8 (69.7) G for NV^-, spin-1 (NV^0, spin-1/2). From Ampere's law, the required current is 87.5 (175) mA, corresponding to 382 (1530) mW considering 50 Ω transmission line ($P = I^2R$).

On the other hand, the Rabi frequency for orbital state can be written as $\Omega_o = d_\perp E_\perp$, where $d_\perp$ is the electric field susceptibility (~1000 kHz/V cm^{-1}), and $E_\perp$ is the electric field. When Ω_o is 100 MHz, $E_\perp$=100 V/cm. When NV is 5um away from both electrode (in between the signal and ground), the required voltage is 0.1 V, corresponding to 200 uW in the 50 Ω transmission line. Thus, ideally, the power suppression more than three orders of magnitude is expected.

In our experiments, the required power for the orbital state manipulation was slightly larger than the above estimation possibly due to the misalignment of the electric field and electric field distribution in the sample. ~500 uW was required to achieve Rabi frequency of 88 MHz (Fig. R9 (a)). Further

decrease of the required power is expected by precise alignment of NV center and electrodes. On the other hand, we have manipulated the spin of NV^- in a different device with the same electrode configuration. To control the spin of NV^- at ~90 MHz Rabi frequency (Fig. R9 (b)), ~300 mW was required at the sample, which was in good agreement with the estimation. Based on the estimation and experimental results, we consider that the argument of the three orders of magnitude increase is reasonable.

We summarized the above calculations in the Supplementary materials as follows. Also, we added “See Supplementary” when we mention the three orders of magnitude difference in the discussion.

[after] Page5, line270-

The required power for Rabi oscillation is hundreds of microwatts, which is three orders of magnitude smaller than that required for spin control using magnetic fields (See Supplementary section III).

[after] Page6, line78- Supplementary materials

¥section{Comparison between electric field control with magnetic field control}

In this section, we compare the efficiency of the electric field orbital control with that of the magnetic field spin control to support the argument in the main text that the electric field orbital control can be performed with three orders of magnitude less power than that required for the magnetic field spin control.

We assume that the NV center is 5 $¥mu$m away from the electrode. The Rabi frequency for the spin is $¥Omega_{¥textrm{s}}=g¥mu_{¥textrm{B}}B_x¥hat{S}_x/¥hbar$, where g is the electron g-factor, $¥mu_{¥textrm{B}}$ is the Bohr magneton, B_x is x-axis magnetic field, and $¥hat{S}_x$ is the spin operator. When $¥Omega_{¥textrm{s}}$ is set to be 100 MHz, B_x must be 34.8 (69.7) G for NV$^-$, spin-1 (NV^0, spin-1/2). From Ampere’s law, the required current is 87.5 (175) mA, corresponding to 382 (1530) mW for a 50 $¥Omega$ transmission line ($P = I^2Z$), where P is power, I is current, Z is impedance.On the other hand, the Rabi frequency for orbital state can be written as $¥Omega_¥textrm{o}=d_{¥perp}E_{¥perp}$, where $d_{¥perp}$ is the electric field susceptibility ($¥sim$1000 kHz/V cm^{-1}), and $E_{¥perp}$ is the electric field. When $¥Omega_¥textrm{o}$ is 100 MHz, $E_{¥perp}$=100 V/cm. When NV is 5 $¥mu$m away from the both electrodes (in between the signal and ground), the required voltage is 0.1 V, corresponding to 200 $¥mu$W in the 50 $¥Omega$ transmission line. Ideally, therefore, power suppression is expected to be more than three orders of magnitude.

In our samples, experimentally, $¥sim$500 $¥mu$W is required to control the orbital state

of NV^0 with the Rabi frequency of $¥sim$90 MHz, while $¥sim$300 mW was needed to control the spin state of NV$^-$ with the Rabi frequency of $¥sim$90 MHz. The required power for the magnetic field control is in good agreement with the above estimation. In contrast, the power required for electric field control is larger than our estimation, which may be due to the displacement and misorientation of NV from the ideal position between the electrodes.

(Comment 3-4)

Minor comments:

- P.1; last paragraph: the subscript (s) is introduced even before the spin itself has been introduced

(Response 3-4)

Thank you for pointing out that. We changed our manuscript as follows.

[before]

(i) Moreover, the subscripts o (s) denote the orbital (spin) degree of freedom.

(ii) Since all experiments are performed under an ambient magnetic field, the transitions from $¥ket{+'}_¥mathrm{o}¥ket{¥uparrow}_¥mathrm{s}$ and $¥ket{-'}_¥mathrm{o}¥ket{¥downarrow}_¥mathrm{s}$ are indistinguishable.

[after]

(i) Page1, line74-

Moreover, the subscripts, o , ~~(s)~~ denotes the orbital ~~(spin)~~ degree of freedom.

(ii) Page2, line97-

Since all experiments are performed under an ambient magnetic field, the transitions from $¥ket{+'}_¥mathrm{o}¥ket{¥uparrow}_¥mathrm{s}$ and $¥ket{-'}_¥mathrm{o}¥ket{¥downarrow}_¥mathrm{s}$ are indistinguishable, where the subscript, s, denotes the spin degree of freedom.

(Comment 3-5)

- P.3; first paragraph: The authors extract ¥lambda = 4.80 GHz from Ref. [26], while there actually a value of 4.9(4)GHz is given. Can the authors comment on this (small) discrepancy?

(Response 3-5)

Sorry for our lacking mention. When we fit the data in Fig. R10 (the inset of Fig. 1(d) in the manuscript), we fitted the data setting λ as a fixed value from 4.0 to 5.0 GHz. The fitting residual (deviation) was almost unchanged from 4.0-4.8 GHz, while the residual increased above 4.9 GHz. 4.8 GHz is the nearest to the reported value (4.9 (4) GHz) among the parameters which showed minimum residual values.

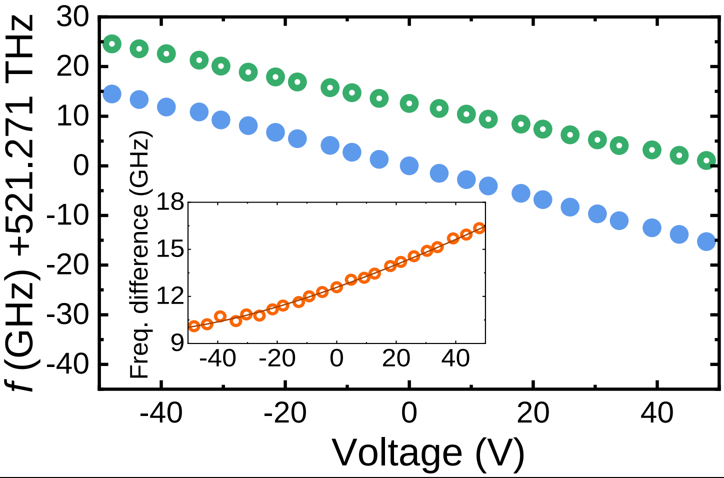

Fig. R10 PLE resonant frequency in NV^0 as a function of the applied voltage.

(Comment 3-6)

- P.4; The concept of (a microwave power dependent) Rabi splitting is hardly introduced and it is not easy to follow. Can the authors extend their explanation? In particular I recommend to introduce the concept of Rabi splitting, where it physically comes from and what it can be used for (e.g. to extract certain parameters).

(Response 3-6)

Thank you for your suggestion. We noticed that the AC Stark splitting or Autler-Townes splitting are more common expression instead of Rabi splitting. Basically, it originates from the formation of the dressed states of the atom due to the strong driving field. Moving on to the rotating frame of the driving field and under the rotating wave approximation, the Hamiltonian of AC driving field can be written as:

$$H/h = {}^{\Omega}/_{2}\, \sigma_x,$$

where the Ω is the Rabi frequency and σ_x is one of the Pauli matrices. By diagonalizing this Hamiltonian, the energy eigenvalues of the resulting field-dressed state become $E/h = \pm {}^{\Omega}/_{2}$ which corresponds to the Autler-Townes splitting (Rabi splitting). We have changed the term and added the description for the main text and theoretical (analytical) explanations for Supplementary information as follows.

[before]

(i) *Using the resonance frequency (12.84 GHz) obtained from the ODER measurement, the microwave power dependence of Rabi splitting is investigated by increasing the microwave power. Figure ¥ref{fig:ODER_ACstark} (c) shows the color map of the PL counts as functions of the relative frequency and square root of the microwave power. The maximum Rabi splitting is 2.6 GHz at 824 $¥mu$W$^{1/2}$. The power dependence of Rabi splitting directly corresponds to the power dependence of the Rabi frequency, which is estimated to be 3.37 MHz/$¥mu$W$^{1/2}$ via fitting.*

(ii) *(c) Rabi splitting as a function of the square root of the power of the applied microwave electric fields.*

[after]

(i) Page4, line 208-

Using the resonance frequency (12.84 GHz) obtained from the ODER measurement, the microwave power dependence of the Autler-Townes splitting is investigated by increasing the microwave power. The Autler-Townes splitting originates from the formation of the color center-field dressed states because of the strong driving field (See Supplementary section I). The splitting between the two peaks corresponds to $d_{¥perp}E_{¥perp}''$, the Rabi frequency. Figure ¥ref{fig:ODER_ACstark} (c) shows the color map of the PL counts as functions of the relative frequency and square root of the microwave power. The maximum Autler-Townes splitting is 2.6 GHz at 824 $¥mu$W$^{1/2}$. The power dependence of Autler-Townes splitting directly corresponds to the power dependence of the Rabi frequency, which is estimated to be 3.37 MHz/$¥mu$W$^{1/2}$ via fitting.

(ii) Figure 3, caption

(c) The Autler-Townes splitting as a function of the square root of the power of the applied microwave electric fields.

(iii) Page4, line53- *" Supplementary materials"*

Also, we briefly mention the derivation of the Autler-Townes splitting in the main text. When we set $¥Delta=0$, $H_{¥text{electric}}^{(¥pm')}/h=d_¥perp E_¥perp'' (¥hat{L}_+ + ¥hat{L}_-)$. After the diagonalization of the Hamiltonian, the resultant energy eigenvalues of the dressed states are $¥pm d_¥perp E_¥perp''/2$. The energy splitting between two dressed state is $d_¥perp E_¥perp''$, corresponding to the Rabi frequency at that driving amplitude. Thus, we can estimate $d_¥perp$ from the energy splitting if we know $E_¥perp''$.

(Comment 3-7)

- P.6; For charge initialization the authors employ very strong 637nm resonant pulses (200uW of power). Can the authors comment on the effect of such strong pulses in relation to spectral diffusion? Are such strong pulses needed, or could also lower power charge initialization be employed, as e.g. used in Ref.[26].

(Response 3-7)

Thank you for your pointing out that. Indeed, strong charge initializing resonant pulses induce spectral diffusion as shown in Fig. R11. When we reduced the initialization power down to 1 uW, the spectral width decreased from ~500 MHz to ~100 MHz (also the readout 575 nm laser is reduced to 100 nW). However, it takes more than 1 ms to initialize the charge state at 1 uW of initialization laser power, while several tens of microseconds (up to 100 us) is required for charge initialization at 200 uW. This long initialization time increases the total measurement time up to several days for a single measurement (e.g. Rabi oscillation at a fixed microwave power). Thus, for the measurements shown in the manuscript, we employed high initialization laser power to shorten the total measurement time.

Of course, it must be better and ideally possible to use low power lasers for charge initialization as in Ref. [R1](=Ref.[26]). However, we do not still have spectrally stable NV center inside a solid-immersion lens (SIL). Without a good SIL structure to enhance the emission from NV center, it was difficult to balance low power charge initialization less than 1 uW with practical measurement time (less than 1 day).

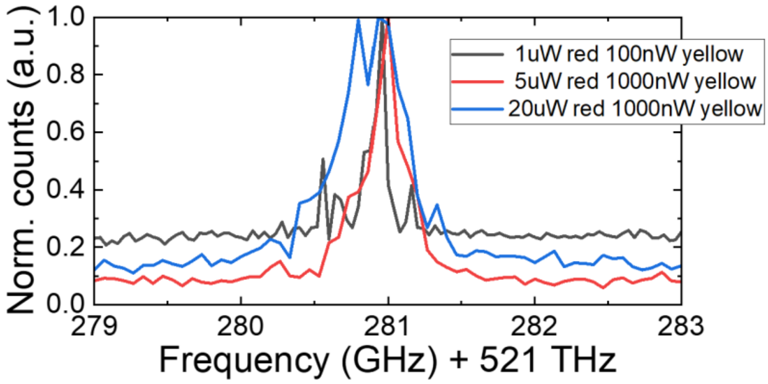

Fig. R11 PLE of NV^0 at different initialization (637 nm) laser power and readout (575 nm) laser power.

[R1] S. Baier et al., PRL 125, 193601 (2020).
https://journals.aps.org/prl/abstract/10.1103/PhysRevLett.125.193601

List of changes (Pages and line numbers are based on the file displaying difference)

Page1, line74-

[before]

Moreover, the subscripts o (s) denote the orbital (spin) degree of freedom.

[after]

Moreover, the subscripts, o , ~~(s)~~ denotes the orbital ~~(spin)~~ degree of freedom.

Page1, line80-

[after]

Under strain, the eigenstates change from $¥ket{¥pm}_{¥text{o}}$ to $¥ket{+'}_{¥text{o}}=¥alpha¥ket{+}_{¥text{o}}+¥beta¥ket{-}_{¥text{o}}$ and $¥ket{-'}_{¥text{o}}=-¥beta¥ket{+}_{¥text{o}}+¥alpha¥ket{-}_{¥text{o}}$ with $¥alpha^2+¥beta^2=1$ (See Supplementaly materials in details).

Page2, line97-

[before] *Since all experiments are performed under an ambient magnetic field, the transitions from $¥ket{+'}_¥mathrm{o}¥ket{¥uparrow}_¥mathrm{s}$ and $¥ket{-'}_¥mathrm{o}¥ket{¥downarrow}_¥mathrm{s}$ are indistinguishable.*

[after] *Since all experiments are performed under an ambient magnetic field, the transitions from $¥ket{+'}_¥mathrm{o}¥ket{¥uparrow}_¥mathrm{s}$ and $¥ket{-'}_¥mathrm{o}¥ket{¥downarrow}_¥mathrm{s}$ are indistinguishable, where the subscript, s, denotes the spin degree of freedom.*

Page2, line105-

[before]

This assumption holds as long as the laser irradiation time is sufficiently short because the lifetime of the spin reaches several milliseconds even under laser irradiation ¥cite{Baier2020}.

[after]

This assumption holds for spin-independent experiments or experiments with time scales shorter than spin relaxation time. Basically, our experiments are assumed to be spin-independent because we can drive and readout both spin states optically and electrically. Furthermore, although reported spin relaxation times range from 570 $¥mu$s to 1.5 s

depending on situations ¥cite{Baier2020, Bradley2022}, they are still longer than the timescale of our experiments ($¥sim$ a few $¥mu$s).

Page4, line 208-

[before] *Using the resonance frequency (12.84 GHz) obtained from the ODER measurement, the microwave power dependence of Rabi splitting is investigated by increasing the microwave power. Figure ¥ref{fig:ODER_ACstark} (c) shows the color map of the PL counts as functions of the relative frequency and square root of the microwave power. The maximum Rabi splitting is 2.6 GHz at 824 $¥mu$W$^{1/2}$. The power dependence of Rabi splitting directly corresponds to the power dependence of the Rabi frequency, which is estimated to be 3.37 MHz/$¥mu$W$^{1/2}$ via fitting.*

[after] *Using the resonance frequency (12.84 GHz) obtained from the ODER measurement, the microwave power dependence of the Autler-Townes splitting is investigated by increasing the microwave power. The Autler-Townes splitting originates from the formation of the color center-field dressed states because of the strong driving field (See Supplementary section I). The splitting between the two peaks corresponds to $d_{¥perp}E_{¥perp}"$, the Rabi frequency. Figure ¥ref{fig:ODER_ACstark} (c) shows the color map of the PL counts as functions of the relative frequency and square root of the microwave power. The maximum Autler-Townes splitting is 2.6 GHz at 824 $¥mu$W$^{1/2}$. The power dependence of Autler-Townes splitting directly corresponds to the power dependence of the Rabi frequency, which is estimated to be 3.37 MHz/$¥mu$W$^{1/2}$ via fitting.*

Page4, line257-

[after]

The origins of the decoherence are attributed ~~to the surrounding nuclear spins ¥cite{Becker2018,Nguyen2019a},~~ spectral diffusion ¥cite{Robledo2010} in the ground state~~,~~ and thermal phonons that are resonant with the orbital transition proposed for SiV ¥cite{Jahnke2015}.

Page5, line271-

[after] -

The required power for Rabi oscillation is hundreds of microwatts, which is three orders of magnitude smaller than that required for spin control using magnetic fields (See Supplementary section III).

Page5, line279-

[after]

If a high impedance superconducting microwave resonator with a zero-point voltage fluctuation of tens of microvolts ¥cite{Samkharadze2016,Niepce2019} is integrated, the single-photon coupling is expected to be tens of kilohertz, allowing further low-power microwave control using several hundred microwave photons. If we can further increase the coupling to $¥sim$1 MHz by bringing the electrode and the color center closer together, we can reach the strong coupling regime of the color center and the microwave resonator, where we can observe single-photon interaction.

Page5, line 289-

[before]

Moreover, controlling the orbital state using phonons is promising. Since the mode volume of the phononic resonator can be designed to be several orders of magnitude smaller than that of typical microwave resonators ¥cite{Neuman2021,Kim2023}, the single-phonon coupling can exceed 1 MHz, allowing the realization of a strong coupling between the single phonon and NV^0.

[after]

Also, we should compare the electric field control of the orbital state with another driving scheme, mechanical driving ¥cite{Maity2020}. The electric field driving requires less complex devices and offers wide frequency range while typical mechanical oscillators employed in experiments are mechanical resonators with narrow frequency range around resonant frequency ¥cite{Chen2018a,Maity2020}. However, though device fabrication processes and frequency tuning result in some more complexity in experiments, controlling the orbital state using phonons is still promising. Since the mode volume of the phononic resonator can be designed to be several orders of magnitude smaller than that of typical microwave resonators ¥cite{Neuman2021,Kim2023}, the single-phonon coupling can exceed 1 MHz without the use

of extremely close proximity electrodes as mentioned above, allowing to reach the strong coupling regime with maintaining both microwave and optical access.

Page 6, line308-

[after]

In addition, a comparison with group IV color centers needs to be discussed. Although the ground state can be modeled with the same Hamiltonian for NV^0 and group IV color centers, considering the spin-orbit coupling, the spin (orbital) Zeeman effect, the strain, and the Jahn-Teller effect which is indistinguishable from strain, the coefficients in each term are different. The largest difference in parameters would be the spin-orbit coupling, which ranges from $¥sim$ 5 GHz in NV^0 to $¥sim$ 400 GHz (corresponding to the splitting of $¥sim$ 800 GHz) in PbV. The ratio of spin-orbit coupling to strain determines the degree of hybridization of the ground state wavefunction, which is essential for magnetic field control of the ground state spin. For NV^0 with 5 GHz spin-orbit coupling, several GHz to tens of GHz of strain parameters are sufficient to fully tune its wavefunction from the spin-orbit coupling dominant regime to the strain dominant regime. In contrast, tens to hundreds of GHz of strain are required to fully control the wavefunction of group IV color centers. Furthermore, we can control the Hamiltonian of NV^0 using dc electric fields, effectively suppressing or enhancing the effect of static strain. Thus, NV^0 can be a good platform to study the effect of strain on the Hamiltonian in a wide parameter range with relatively small strain parameters and dc electric fields. It should be noted that the excited state Hamiltonian of NV^0 and group IV color centers are different. While NV^0 has two optically excited states with two spin degrees of freedom and one orbital degree of freedom, group IV color centers have optically excited states with two spin degrees of freedom and two orbital degrees of freedom. This difference may affect the readout scheme when considering the single-photon readout demonstrated in Ref. ¥cite{Nguyen2019}.

Figure

Figure 5, caption

[before]

The blue dots represent the data and the blue curve represents the fitting using the function, $A¥mathrm{sin}(¥omega t+¥phi)¥mathrm{exp}(-t/T_2^*)+B$, where A, B, and $¥phi$ are constants, $¥omega$ is the angular frequency, T_2^* is the orbital coherence time. T_2^* is 30.2 ns.

[after]

The blue dots represent the data and the blue curve represents the fitting using the function, $A¥mathrm{sin}(¥Delta t+¥phi)¥mathrm{exp}(-t/T_2^*)+B$, where A, B, and $¥phi$ are constants, $¥Delta$ is the detuning from the transition between $¥ket{0}$ and $¥ket{1}$, and T_2^* is the orbital coherence time. T_2^* is 30.2 ns.

Figure 2, 3, 4, 5

Add error bars

Figure 2, 4, 5 caption

Add the sentence “Error bars correspond to standard deviation error.”

Figure 3, caption

[before]

(c) Rabi splitting as a function of the square root of the power of the applied microwave electric fields.

[after]

The Autler-Townes splitting as a function of the square root of the power of the applied microwave electric fields.

Method

Page6 line 389

[after]

The charge initialization fidelity is estimated to be $>97.0¥%$ from the residual fluorescence after the irradiation of long charge initialization pulse.

Supplementary materials

Page2, line 20-

[before]

Because NV^0 has the same wave function symmetry as NV$^-$, the effects of the strain and electric fields on $¥ket{e_{x,y}}_{¥text{o}}$ are considered to be similar to that in the excited state of NV$^-$ ¥cite{Maze2011}.

[after]

Because NV^0 has the same wave function symmetry as the excited state of NV$^-$ due to the dynamic Jahn-Teller effect (the effective C_{3v} symmetry), the effects of the strain and electric fields on $¥ket{e_{x,y}}_{¥text{o}}$ are considered to be similar to that in the excited state of NV$^-$ ¥cite{ Gali2009,Maze2011,Zhang2011}.

Page4, line 48

[after]

Subsequently, considering the sinusoidal driving fields, we move to the rotating frame with $H_¥text{rot}/h=f_¥text{d}¥hat{L}_z/2$ from the system Hamiltonian, $¥sqrt{¥lambda^2+¥epsilon_{¥perp}^2} ¥hat{L}_z+H_{¥text{electric}}^{(¥pm')}/h$.

Page4, line53-

[after]

Also, we briefly mention the derivation of the Autler-Townes splitting in the main text. When we set $¥Delta=0$, $H_{¥text{electric}}^{(¥pm')}/h=d_¥perp E_¥perp" (¥hat{L}_+ + ¥hat{L}_-)$. After the diagonalization of the Hamiltonian, the resultant energy eigenvalues of the dressed states are $¥pm d_¥perp E_¥perp"/2$. The energy splitting between two dressed states is $d_¥perp E_¥perp"$, corresponding to the Rabi frequency at that driving amplitude. Thus, we can estimate $d_¥perp$ from the energy splitting if we know $E_¥perp"$.

Page6, line78-

[after]

¥section{Comparison between electric field control with magnetic field control}

In this section, we compare the efficiency of the electric field orbital control with that of the magnetic field spin control to support the argument in the main text that the electric field

orbital control can be performed with three orders of magnitude less power than that required for the magnetic field spin control.

If we assume that the NV center is 5 $¥mu$m away from the electrode, The Rabi frequency for the spin is $¥Omega_{¥textrm{s}}=g¥mu_{¥textrm{B}}B_x¥hat{S}_x/¥hbar$, where g is the electron g-factor, $¥mu_{¥textrm{B}}$ is the Bohr magneton, B_x is x-axis magnetic field, and $¥hat{S}_x$ is the spin operator. When $¥Omega_{¥textrm{s}}$ is set to be 100 MHz, B_x must be 34.8 (69.7) G for NV$^-$, spin-1 (NV^0, spin-1/2). From Ampere's law, the required current is 87.5 (175) mA, corresponding to 382 (1530) mW for a 50 $¥Omega$ transmission line ($P = I^2Z$), where P is power, I is current, Z is impedance.On the other hand, the Rabi frequency for orbital state can be written as $¥Omega_¥textrm{o}=d_{¥perp}E_{¥perp}$, where $d_{¥perp}$ is the electric field susceptibility ($¥sim$1000 kHz/V cm^{-1}), and $E_{¥perp}$ is the electric field. When $¥Omega_¥textrm{o}$ is 100 MHz, $E_{¥perp}$=100 V/cm. When NV is 5 $¥mu$m away from the both electrodes (in between the signal and ground), the required voltage is 0.1 V, corresponding to 200 $¥mu$W in the 50 $¥Omega$ transmission line. Ideally, therefore, power suppression is expected to be more than three orders of magnitude.

In our samples, experimentally, $¥sim$500 $¥mu$W is required to control the orbital state of NV^0 with the Rabi frequency of $¥sim$90 MHz, while $¥sim$300 mW was needed to control the spin state of NV$^-$ with the Rabi frequency of $¥sim$90 MHz. The required power for the magnetic field control is in good agreement with the above estimation. In contrast, the power required for electric field control is larger than our estimation, which may be due to the displacement and misorientation of NV from the ideal position between the electrodes.

Other minor changes

Page2, line126

[before] See Supplementary materials for detail

[after] See Supplementary section I

Page3, line138-

[before] See Supplementary materials for detail

[after] See Supplementary section II

Page3, line 146

[begore] 363

[after] 348 (12)

Page3, equation (3)

Divide the 1st and 2nd terms by 2.

Page3, line183

Multiply the first term or the equation in the line by 2.

Page3, lin186

[before] See Supplementary materials for detail

[after] See Supplementary section I

Page3, line 164 T1

[before] 137

[after] 138 (19)

Page4, line 206 FWHM ODER

[before] 130

[after] 133 (10)

Page4, line223

[before] 961 kHz

[after] 1.0 MHz

Page4, line225

[before] See Supplementary materials for detail

[after] See Supplementary section II

Page4, line 223-230

[before] ¥epsilon

[after] $d_{¥perp}^{¥mathrm{AC}}$

Page4, line226

[before] 363 kHz

[after] 348 kHz

Page4, line239-

[before] 828

[after] 504

Page4, line 247 slope of Rabi frequency

[before] 3.86

[after] 3.86 (3)

Page4, line 257 T2*

[before] 30.2

[after] 31.0 (3.6)

Page6, line 345 T1

[before] 137

[after] 138

Supplementary, Page2, equation(13)

Divide the 1st and 2nd terms by 2.

Supplementary, Page2 line52

Multiply the first term of the equation in the line by 2.

Supplementary, equation (11) and (12)

Add (t) to the electric field to indicate that electric fields are assumed to be time-dependnt.

REVIEWERS' COMMENTS

Reviewer #1 (Remarks to the Author):

Upon reviewing the revised version of the manuscript [Title or ID] and the authors' response to my comments, I am satisfied with the changes made.

Reviewer #2 (Remarks to the Author):

The authors have responded to all of my concerns and I am happy to recommend publication in Nat. Comms

Reviewer #3 (Remarks to the Author):

The authors have properly addressed all my comments, as well gave very detailed explanations to all the question I have raised. I agree with their assessment on (eventually shorter) spin lifetime, appreciate that error bars have been added and that now details on the required power level for electric field driving are discussed. I also thank the authors for their detailed answers to my minor comments.

One point I would like to raise again concerns Answer 3-5: The way the authors do this fitting is still not clear from the manuscript. Either they leave \lambda as a free fitting parameter and discuss the (possible) difference from the literature value. Or they fix it to the literature value. Fixing it (somewhat) close to the literature value is not a rigor method. I leave the choice between the two options to the authors. I suggest that in either case they add a sentence to the manuscript explaining how the literature value \lambda is treated.

It should also be mentioned that the author’s detailed response to the comments of the other referees helped to further improve the quality of the manuscript. I can support publication of this manuscript in Nature Communications.

List of changes

We made a change shown below responding to the comment from Reviewer#3. We believe this change does not influence results and discussions in the manuscript.

Comment from Reviewer #3

One point I would like to raise again concerns Answer 3-5: The way the authors do this fitting is still not clear from the manuscript. Either they leave ¥lambda as a free fitting parameter and discuss the (possible) difference from the literature value. Or they fix it to the literature value. Fixing it (somewhat) close to the literature value is not a rigor method. I leave the choice between the two options to the authors. I suggest that in either case they add a sentence to the manuscript explaining how the literature value ¥lambda is treated.

Page3, left column

(before)

The frequency differences are then fitted using the equation $2¥sqrt{¥lambda^2+(¥epsilon_{¥perp}+d_{¥perp}E_{¥perp})^2+(d_{¥perp}E_{¥perp}')^2}$ and a fixed $¥lambda=4.80$ GHz ¥cite{Baier2020}. Consequently, the estimated parameters are $¥epsilon_{¥perp}$ = 4.06 GHz and $d_{¥perp}$ = 348 (12) kHz/(V cm^{-1}).

(after)

The frequency differences are then fitted using the equation $2¥sqrt{¥lambda^2+(¥epsilon_{¥perp}+d_{¥perp}E_{¥perp})^2+(d_{¥perp}E_{¥perp}')^2}$ and a fixed $¥lambda=4.90$ GHz based on the value in Ref. ¥cite{Baier2020}. Consequently, the estimated parameters are $¥epsilon_{¥perp}$ = 3.92 GHz and $d_{¥perp}$ = 358 (14) kHz/(V cm^{-1}).

Page4, right column

(before)

Additionally, the electric susceptibility is estimated to be $d_{¥perp}^{¥mathrm{AC}}=$ 1.0 MHz/(V cm^{-1}) (See Supplementary section II). Here, $d_{¥perp}^{¥mathrm{AC}}$ is larger than that obtained from the measurement using DC electric fields (348 kHz/(V cm^{-1})). The difference can be attributed to electric field screening during DC measurement, which decreases the effective electric fields at the NV center ¥cite{Block2021a}.

(after)
Additionally, the electric susceptibility is estimated to be $d_{¥perp}^{¥mathrm{AC}}=$ 1.0 MHz/(V cm^{-1}) (See Supplementary section II). Here, $d_{¥perp}^{¥mathrm{AC}}$ is larger than that obtained from the measurement using DC electric fields (358 kHz/(V cm^{-1})). The difference can be attributed to electric field screening during DC measurement, which decreases the effective electric fields at the NV center ¥cite{Block2021a}.

Minor changes

We noticed that some of the term “Rabi splitting” remain unchanged. We unified to the same notation, “the Autler-Townes splitting” as follows.

Page 3 Subsection title
(before) Optically-detected electrical resonance and Rabi splitting
(after) Optically-detected electrical resonance and the Autler-Townes splitting

Caption of Fig.3
(before)
¥caption{Optically-detected electrical resonance (ODER) and Rabi splitting measurements. (a) Schematic of the experimental sequence used in measuring the ODER and Rabi splitting. After charge conversion using the 637-nm laser, the 575-nm laser is simultaneously applied with microwave application at the transition between $¥ket{0}$ and $¥ket{2}$. The frequencies of the 575-nm laser are then swept for ODER measurement, and the power of the microwave is swept for the measurement of Rabi splitting. (b) ODER spectrum of NV^0. The orange dots represent the data and the red curve is the Gaussian fit. (c) Rabi splitting as a function of the square root of the power of the applied microwave electric fields.

(after)
¥caption{Optically-detected electrical resonance (ODER) and the Autler-Townes splitting measurements. (a) Schematic of the experimental sequence used in measuring the ODER and the Autler-Townes splitting. After charge conversion using the 637-nm laser, the 575-nm laser is simultaneously applied with microwave application at the transition between $¥ket{0}$ and $¥ket{2}$. The frequencies of the 575-nm laser are then swept for ODER measurement, and the power of the microwave is swept for the measurement of the Autler-Townes splitting. (b) ODER

spectrum of NV^0. The orange dots represent the data and the red curve is the Gaussian fit. (c) The Autler-Townes splitting as a function of the square root of the power of the applied microwave electric fields.

Page 3, right column

(before) When $¥Delta¥sim0$, the second term that remains in Equation (¥ref{Hamiltonian-electric}) contributes to Rabi splitting and Rabi oscillation, which will be discussed in below.

(after) When $¥Delta¥sim0$, the second term that remains in Equation (¥ref{Hamiltonian-electric}) contributes to the Autler-Townes splitting and Rabi oscillation, which will be discussed in below.

Page 4, right column

(before) Here, the Rabi frequency linearly increases with the square root of the microwave power, and the slope is 3.86 (3) MHz/$¥mu$W$^{1/2}$, which is in good agreement with the value obtained from the Rabi splitting measurement of 3.37 MHz/$¥mu$W$^{1/2}$.

(after) Here, the Rabi frequency linearly increases with the square root of the microwave power, and the slope is 3.86 (3) MHz/$¥mu$W$^{1/2}$, which is in good agreement with the value obtained from the Autler-Townes splitting measurement of 3.37 MHz/$¥mu$W$^{1/2}$.

We noticed that we must calculate the power using the root-mean-square current (voltage) in Section III, Supplementary Information. We recalculate the power required for the qubit state control. These changes do not affect our discussions.

Supplementary Information, section III

(before1) From Ampere's law, the required current is 87.5 (175) mA, corresponding to 382 (1530) mW for a 50 $¥Omega$ transmission line ($P = I^2Z$), where P is power, $I_¥textrm{rms}$ is current, Z is impedance.

(after1) From Ampere's law, the required current amplitude is 87.5 (175) mA ($I_¥textrm{rms} = 87.5 (175) ¥times 1/¥sqrt{2}$ mA), corresponding to 191 (765) mW for a 50 $¥Omega$ transmission line ($P = I_¥textrm{rms}$^2Z$), where P is power, $I_¥textrm{rms}$ is the root-mean-square current, Z is impedance.

(before2) When NV is 5 $¥mu$m away from the both electrodes (in between the signal and ground), the required voltage is 0.1 V, corresponding to 200 $¥mu$W in the 50 $¥Omega$ transmission line. Ideally, therefore, power suppression is expected to be more than three orders of magnitude.

(after2) When NV is 5 $¥mu$m away from the both electrodes (in between the signal and ground), the required voltage amplitude is 0.1 V ($V_¥textrm{rms}=0.1¥times1/¥sqrt{2}$ V), corresponding to 100 $¥mu$W in the 50 $¥Omega$ transmission line. Ideally, therefore, power suppression is expected to be more than three orders of magnitude.

We changed sentences in Supplementary Information for clarity.

Supplementary Information, section IA

(before) For the strain Hamiltonian, the axis of rotation can be redefined for simplicity and expressed as follows:

(after) By redefining the axis of rotation, the strain Hamiltonian can be expressed as follows:

Caption of Fig. 3

(before)

The frequencies of the 575-nm laser are then swept for ODER measurement, and the power of the microwave is swept for the measurement of the Autler-Townes splitting.

(after)

The microwave frequencies are then swept for ODER measurement, and the power of the microwave is swept for the measurement of the Autler-Townes splitting.

Additionally, we added some minor changes regarding to the capital letters (like dc or DC) and the order in the equations.
